# ER-depletion lowering the 'hypothalamus-uterus-kidney' axis functions by perturbing the renal ERβ/Ptgds signalling pathway

**DOI:** 10.18632/aging.102401

**Published:** 2019-11-10

**Authors:** Yan-Ru Liu, Zhi-Shu Tang, Jin-Ao Duan, Lin Chen, Jing Sun, Rui Zhou, Zhong-Xing Song, Xin-Bo Shi, Hui-Yuan Zhu

**Affiliations:** 1 Shaanxi Province Key Laboratory of New Drugs and Chinese Medicine Foundation Research, Shaanxi Collaborative Innovation Center of Chinese Medicinal Resources Industrialization, Shaanxi University of Chinese Medicine, Xianyang 712083, P.R. China; 2 Key Laboratory for High Technology Research of TCM Formulae and Jiangsu Collaborative Innovation Center of Chinese Medicinal Resources Industrialization, Nanjing University of Chinese Medicine, Nanjing 210023, P.R. China; 3 Shaanxi University of Chinese Medicine, Xianyang 712083, P.R. China

**Keywords:** estrogen receptor beta, ‘hypothalamus-uterus-kidney’ axis, prostaglandin D synthase _2_, menopause

## Abstract

Researchers have long assumed that systematic estrogen fading might contribute to the sustained progression of menopausal degenerate syndromes, although definitive evidence has not been presented. Whether such findings represent a causal contribution or are the result of opportunistic messengers sent from the reproductive system to the brain is also a vital question. We constructed a multiscale network of the ovariectomy (OVX) induced estrogen receptors depletion (ER-depletion) model and integrated targeted proteomic, targeted lipidomic, cytochemical, and histopathological data across three tissues from the ovariectomy rodent model. We found that compared to control rats, OVX rats showed increased renal and uterine prostaglandin D2 synthase (Ptgds) expression and decreased hypothalamic Ptgds expression, abnormal Ptgds metabolites, the degenerate renal function profiles and decreased cognitive ability (learning and memory) in Morris water maze test. Importantly, we observed a regulatory relationship among ER (particularly ERβ), the degree of the pathological phenotype, learning behavior test and the ‘hypothalamus-uterus-kidney (HUK) axis functions. Collectively, this study elucidates that ER depletion promoted HUK aging is mostly attributed to a renal ERβ/Ptgds signalling imbalance.

## INTRODUCTION

Female ageing begins with the ageing of the reproductive system, which drives primordial follicular ovarian pools to accelerate consumption. Accompanied by estrogen depletion and turbulence, menopausal ovarian failure is the final step in this process [1]. Menopause, an inevitable stage of ageing among 45~55-year-old women, is a complex process involving a variety of cellular and molecular changes. Menopause has been described to have different “appearances”; the main physiological manifestations are ovary fading and suffering from various phenotypic syndromes, such as hot flashes, anxiety, insomnia, depression, osteoporosis, and cognitive decline [2]. In addition to the typical symptoms of these menopausal syndromes, there is actually a symptom of rapid ageing. The emergence of rapid ageing is partially due to age, and rapid ageing has a very important relationship with the loss of estrogen in menopause.

Data from epidemiological studies show that menopausal women have a higher risk to develop chronic kidney disease (CKD) [3]. These conditions will not occur spontaneously in the same individual but may subsequently lead to related neurological diseases. Patients with kidney small vessel disease, endothelial dysfunction, and increased oxidative stress are prone to CKD, which induces cognitive impairment in patients [4]. A higher prevalence ranging from 16% to 38% of cognitive decline is related to CKD and increases the risk of mortality [5]. Due to a lack of updated guidelines or commentary for menopausal women with clinical nephrological diseases, research about the relevance of ovarian failure-induced estrogen loss on CNS dysfunction and the mechanistic links between the dysfunction of the kidney and brain also needs to be conducted. Considering previous results, we found that many menopause symptoms are significantly associated with elevated renal toxicity markers [6]. The results of these documents are suggestive of a nephrology contribution to menopausal degenerate function, though reports offer little insight into promising mechanisms, and a consistent relationship with specific renal genes has not been explored. Therefore, before revealing the ovarian failure response to the HUK axis, understanding and exploring the relationship between the effects of menopausal oestrogen and kidney disease is more essential. Since estrogen binds to nuclear estrogen receptors to alter the expression of many genes in the modulation of the body’s systemic metabolism, it is necessary to identify estrogen response genes and to explore their binding interactions before evaluating the risks of estrogen loss on organs in menopause. Certainly, it is unrealistic to explore how the multiple genes and their signalling pathways act on organs in a parallel way. Recent findings have indicated that a higher prevalence of renal lipotoxicity was observed among a menopausal CKD group. As an important factor in the development of impaired renal function in menopausal women, lipotoxicity can stimulate chronic inflammation and exacerbate oxidative stress. As pro-inflammatory adipose tissue macrophages (ATMs) accumulate, the resultant lipid imbalance may trigger macrophage infiltration and pro-inflammatory cytokine production, thus leading to local fat cell dysfunction [7].

Here, we conducted this study to draw and match biological networks underlying two distinct menopausal-related phenotypes via multiple independent datasets collected from rodents. We assumed that a multiscale network of ovariectomized (OVX) models would provide fresh insight into the molecular endocrinology context without clinical symptoms [8]. By using OVX rats as a model of systemic disruption, we began with a computational characterization of a specific endophenotype of menopause. Then, we directly enriched specific genes and metabolites in a multiscale network calculation of multiomic datasets together with biochemistry, histopathology, immunohistochemistry, Western blotting, and mRNA sequencing data. The exhaustion of the female reproductive system and the kidney function alterations with fluctuating levels of lipid metabolic genes affects the systemic metabolism. This study maps a ‘disease phenotypes-genes- metabolites’ network to explore whether ER-depletion induced renal profile disturbing results in brain dysfunction. The study design offers novel evidence in a data-driven approach to link renal lipid-gene distortion in menopause period, which facilitates the integration of diverse biomedical data with multiple disease stages. Our results provide evidence of renal gene perturbations in menopausal degenerative diseases, particularly involved in lipid metabolism pathways. In summary, these data provide compelling evidence linking the relationship between ER-depletion with renal lipid gene along the ‘Hypothalamus-uterus-kidney’ axis after ovarian failure.

## RESULTS

### OVX-induced ER-depletion promotes distinct proteomic signalling outcomes

**Figure 1:**
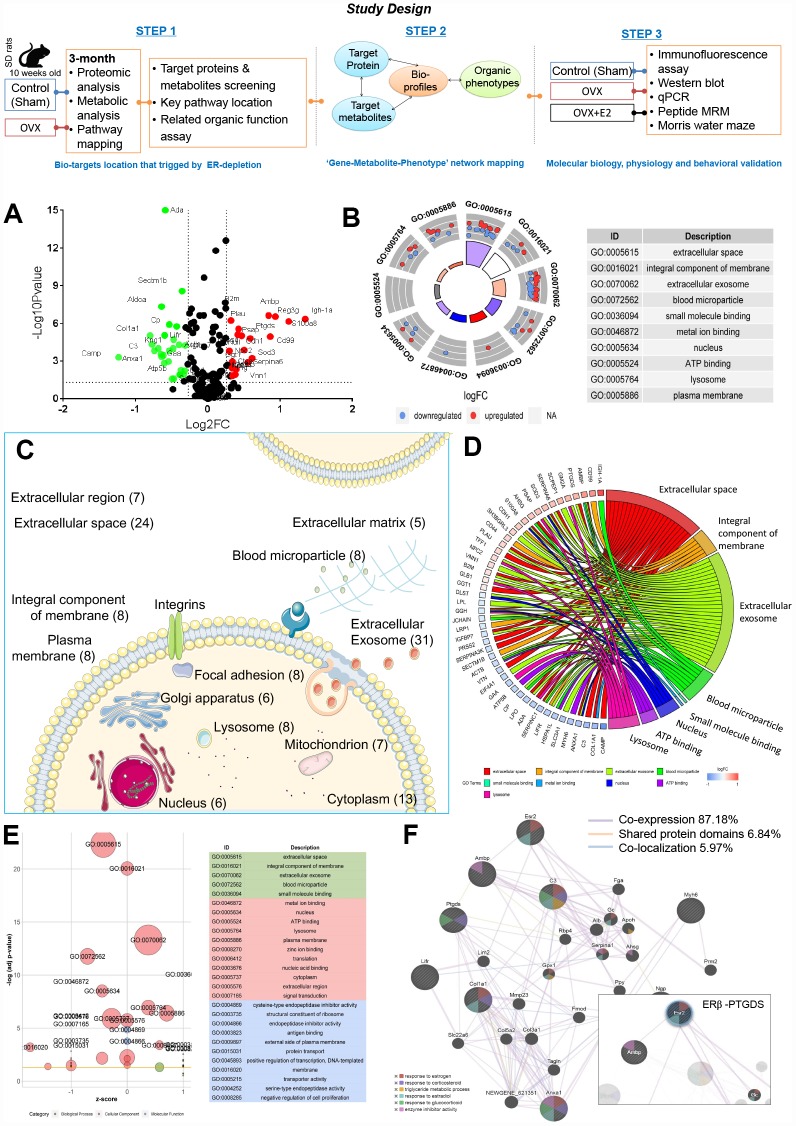
**Ovarian failure induced overall proteomic regulation dysfunction as shown by proteomic analysis.** (**A**) Volcano plot of differential proteins screened between control and OVX rats (normalized by control sample data). (**B**) GO Circle plot for significant enrichment of cellular components (CC) for genes that are strong candidates for ovarian failure-induced dysfunction. (**C**) Subcellular locations of ovarian failure proteins annotated by Gene Ontology. (**D**) GO-Chord plot illustrating the gene-annotation enrichment relationships and representative significant GO terms that are distinctly classified into species-specific gene clusters. (**E**) GO-Bubble plot illustrating significant enrichment of molecular functions and biological processes (adjusted p-value ˂ 0.05) and the z-score of the term (**F**) Protein–protein interaction network identified using GeneMANIA (direct interaction database).

The experimental protocol was shown in Figure 1 (Study design). Since the proteome provides extensive information on the molecular mechanisms that regulate the cellular events between genotypes and their phenotypic manifestations [9]. To understand the underlying ovarian failure induced ER-depletion process on systemic function, we performed an isobaric tags for relative and absolute quantification (iTRAQ)-based proteomics analysis on urine samples from control (sham) and ovariectomized (OVX) rats.

Among the global proteins in the rat proteome, 251 proteins were quantified. We then selected differentially expressed proteins based on the cut-off conditions of a 1.5-fold change and a p value ˂ 0.05. As a result, 55 proteins were identified to be highly related and differentially expressed, with 25 upregulated and 30 downregulated proteins, triplicate analysis was performed (Figure 1A, Supplementary Table 3).

Ingenuity pathway analysis (IPA) identified ‘Neurological Disease’ (p = 3.61E-09 to 4.26E-03, 22 proteins), ‘Reproductive System Disease’ (p = 1.23E-04 to 2.98E-03, 20 proteins), ‘Metabolic Disease’ (p = 1.52E-04 to 4.26E-03, 17 proteins), ‘Renal and Urological System Development and Function’ (p = 1.56E-04 to 1.86E-03, 4 proteins), and ‘Renal and Urological Disease’ (p = 4.59E-04 to 2.13E-03, 5 proteins) (Supplementary Table 4) as the most significant disease categories. Interestingly, within these categories, the most significantly associated upstream regulator term was ‘beta-estradiol’ (p = 1.06E-08), which was associated with ovarian failure-induced abnormalities. Notably, organic toxicity functions were most significantly associated with ‘Acute Renal Failure Panel’ (p = 9.18E-06, 4 proteins), ‘Long-term Renal Injury Antioxidative Response Panel’ (p = 6.66E-04, 2 proteins), ‘Positive Acute Phase Response Proteins’ (p = 1.86E-03, 2 proteins), and ‘Persistent Renal Ischemia-Reperfusion Injury’ (p = 1.86E-03, 2 proteins) (Supplementary Table 5). These proteome changes altered by OVX indicated a dramatic effect on the estrogen regulation and more intensive occurred in kidney.

### ER-depletion disturbs the cellular communication in renal microenvironments

All differentially expressed proteins were mapped to various terms in the Gene Ontology (GO) database, and the proteins for each term were counted and calculated by a hypergeometric test and were mapped to the enriched GO terms. GO annotation indicated that the ovarian failure proteome was significantly enriched in the ‘Cellular Components of the Extracellular Space’, ‘Extracellular Exosome’, ‘Extracellular Space’, ‘Integral Component of the Membrane’, ‘Blood Microparticle’, ‘Nucleus’, ‘Lysosome’, ‘Plasma Membrane’, ‘Cytoplasm’, and ‘Extracellular Region’ (Figure 1B, Supplementary Table 3). In particular, many secreted proteins were annotated as ‘Extracellular Exosomes’ (28 proteins, including 14 upregulated and 14 downregulated proteins) or ‘Extracellular Space’ (22 proteins, including 10 upregulated and 12 downregulated proteins) (Figure 1C, 1D, Supplementary Table 3). Notably, among these abnormally expressed proteins, 19 proteins were reported as kidney disease markers (Ada, Ambp, Anxa1, B2m, Camp, Col1a1, Cp, Ggt1, Glb1, Lgfbp7, Lifr, Lpl, Plau, Ptgds, S100a8, Serpinc1, Serpina3k, Tff1, and Vtn) [10–25] (Supplementary Table 3, highlight in green). These data suggest that ER depletion mainly interferes renal proteins’ extracellular communication.

### ER-depletion alters renal lipid molecular transport functions

According to IPA disease categories, molecular function (MF) and biological process (BP) enrichment of these 19 renal-specific proteins exhibited major lipid metabolism (4.41E-06 to 4.09E-03, 5 proteins, Supplementary Table 6), including calcium ion binding (Anxa1, S100a8) lipoprotein particle receptor activity (Lpl), prostaglandin-D synthase activity (Ptgds), acyl-glycerol metabolic process (Lpl), neutral lipid metabolic process (Lpl), response to lipopolysaccharide (S100a8, Plau, Serpina1,), response to corticosteroid (Col1a1, Anxa1, Ptgds), lipid transport (Anxa1), fatty acid biosynthetic process (Anxa1, Ptgds, Lpl), response to glucocorticoid (Ptgds, Anxa1), and lipid localization (Anxa1) involving in7 proteins including Anxa1, Col1a1, Lpl, Plau, Ptgds, S100a8, and Serpinc1 (Supplementary Table 3, highlight in yellow). These 8 proteins, renal-specific proteins annotated in the Human Protein Atlas were significantly dysregulated, revealing that the loss of renal lipid protein is an important overall feature of ovarian failure (Figure 1E). These observations indicate that diminishing ovarian estrogen secretion triggers extensive renal lipid metabolism derangement.

### Ovarian failure leads to enrichment of the signalling pathways associated with ERβ-regulated cell-to-cell signalling and renal lipid metabolism

Network analysis returned three correlation networks linking all of the differentially regulated proteins with an IPA p-score (IPA p-score = −log10 [p value]), including ‘Cellular Movement, Haematological System Development and Function, Immune Cell Trafficking’ (IPA p-score = 42, 18 proteins), and ‘Cell-To-Cell Signalling and Interaction, Carbohydrate Metabolism, and Lipid Metabolism’ (IPA p-score = 11, 7 proteins). Consistent with the results of the previous analysis, eight proteins implicated in renal lipid metabolic disease have been highlighted to be associated with the network, including upregulated Ptgds, Ambp and downregulated Anxa1, Camp, Col1a1, Lifr, and Myh6 (Supplementary Tables 7 and 8).

We then further examined the eight proteins’ relationships with estrogen regulation and explored the proteins with the greatest impacts on ovarian failure using the GeneMANIA platform. All eight proteins were subjected to leading edge analysis by physical interactions, shared protein domains, and co-localization. The weighted results showed that the six terms that were most significantly associated with the biological process closely related to estrogen-regulated renal lipid metabolism at a cut-off FDR ˂ 10% were ‘Response to Estrogen’, ‘Response to estradiol’, ‘Triglyceride Metabolic Process’, ‘Response to Glucocorticoid’, ‘Response to Corticosteroid’, and ‘Enzyme Inhibitor Activity’ (Figure 1F, Supplementary Table 9). Meanwhile, we found a close co-expression correlation among ERβ, Ambp, Ptgds, Anxa1, and Col1a1 in the network.

Furthermore, Kyoto Encyclopedia of Genes and Genomes (KEGG) enrichment from MetaboAnalyst platform (http://www.metaboanalyst.ca) [26] returned two metabolism pathways including ‘Arachidonic acid (AA) metabolism’ (p=0.003, path: rno00590, Ptgds, Ggt1), and ‘Taurine and hypotaurine metabolism’ (p=0.009, path: rno00430, Ggt1). In line with the KEGG annotation, Ptgds was highlighted as being associated with the lipid metabolism networks (Supplementary Table 10). These findings suggest that the gene association sets of ERβ/Ptgds singling pathway may have close relationships with lipid synthesis, transfer, and metabolic signalling between extra- and intracellular membranes.

### Urinary and serum eicosanoid disturbances reflect lipid metabolism disorder

Eicosanoids in arachidonic acid metabolism are key metabolites in the aetiology of metabolic syndrome (MetS), we next investigated whether the eicosanoid perturbation in rats with ovarian failure was due to the ER-depletion and defects in renal absorption. Therefore, we measured local metabolites in the kidney by metabolome analysis (Supplementary Figure 2).

**Figure 2:**
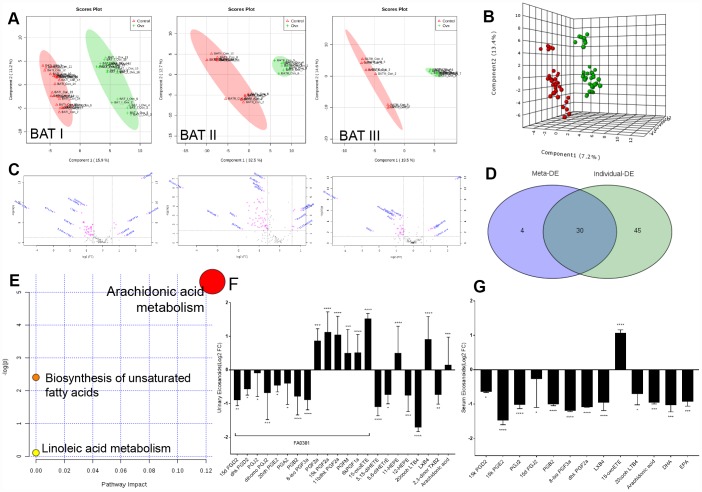
**Imbalances of eicosanoids in the kidneys of OVX rats.** (**A**) PLS analysis of the metabolome profile of the kidney in the control and OVX rats from three experiments. (**B**) Meta-analysis and PLS analysis of urinary eicosanoids in control rats (n = 45) and OVX rats (n = 45). (**C**) Volcano plot for eicosanoid changes with pink colour reflecting decreases/increases. (**D**) Venn diagram of the top differentially expressed features from the meta-analysis. (**E**) Pathway enrichment plot of the biomarkers in each pathway. (**F**) Liquid chromatography–tandem mass spectrometry analysis of eicosanoids (Log2 FC) from urine samples. (**G**) Liquid chromatography–tandem mass spectrometry analysis of eicosanoids (Log2 FC) from serum samples. n=6, mean ± s.d., compared to control rats, *p < 0.05, **p < 0.005, ***p < 0.0005 and ****p < 0.0001.

PLS (partial least-square) analysis from MetaboAnalyst platform of the metabolome showed a distinct change in the metabolite profiles in the kidneys of rats with ovarian failure 12 weeks after ovariectomy relative to those of the age-matched control rats as represented by substantial eicosanoids distortion in the urinary system (three experiments, Figure 2A). Here, significant features were selected based on a specific criterion with a cut-off adjusted p value < 0.05, a fold change ˃ 1.5, and an AUC ˃ 0.75 for each dataset (Figure 2B). Statistical analysis of the metabolomics data from the OVX and control rats revealed statistically significant differences in ions between these two groups, as shown in the volcano plot (Figure 2C). Average variable importance in projection (VIP) scores was also used to calculate feature importance with a VIP score ˃ 1. After meta-analysis selection, 22 and 13 of 155 eicosanoids in the urine and serum, respectively, were identified as potential biomarkers for arachidonic acid metabolism evaluation (Figure 2D, Supplementary Tables 3, 11 and Supplementary Figure 1). Pathway enrichment analysis of the KEGG database indicated that these 35 eicosanoid markers were mostly involved in the following three metabolic pathways: AA metabolism, biosynthesis of unsaturated fatty acids, and linoleic acid metabolism (Figure 2E).

Pathway enrichment analysis detected that the most significant differences between the OVX rats and the control rats were related to prostaglandins. As major products of AA generated from mast cells, the physiological importance of prostaglandins is illustrated by the numerous diseases to which these abnormalities contribute, including inflammatory diseases, diabetes, obesity, and cancer. The abundant prostaglandins in urine that exhibited rapid decreasing trends included 15d PGD_2_, dhk PGD_2_, PGJ_2_, dihomo PGJ_2_, 20oh PGE_2_, PGA_2_, PGB_2_, and 8-iso PGF_3a_, whereas AA, PGF_2a_, 15k PGF_2a_, 11bdhk PGF_2a_, PGFM, and 6kPGF_1a_ showed increasing trends. Moreover, a significant reductions in the levels of prostaglandins in the serum, such as 15k PGE_2_, 8-iso PGF_3a_, dhk PGF_2a_, PGJ_2_, PGB_2_, 15k PGD_2_, and 15d PGJ_2_, as well as the depletion of the unsaturated fatty acids AA, docosahexaenoic acid (DHA), and eicosapentaenoic acid (EPA) (Figure 2F, 2G; Supplementary Table 11). These prostaglandin alteration trends, which correspond to the proteomic data, also indicated weakened adipose cell transportation in rats with ovarian failure induced ER-depletion, which attenuates renal lipid metabolism and ultimately promotes local lipid accumulation.

### The ‘Gene-Metabolite-Phenotype’ network revealed ER-depletion trigger Ptgds abnormal expression and perturbs HUK axis functions

To study complex ER-depletion induced metabolism disease and to gain systemic functional insights, we evaluated Ptgds activity in a multiscale network analysis via SIMCA-p (version 14.0, Umetrics, Umeå, Sweden) of four datasets containing organic phenotype datasets, organic function datasets, key protein datasets, and metabolomic datasets.

**Figure 3:**
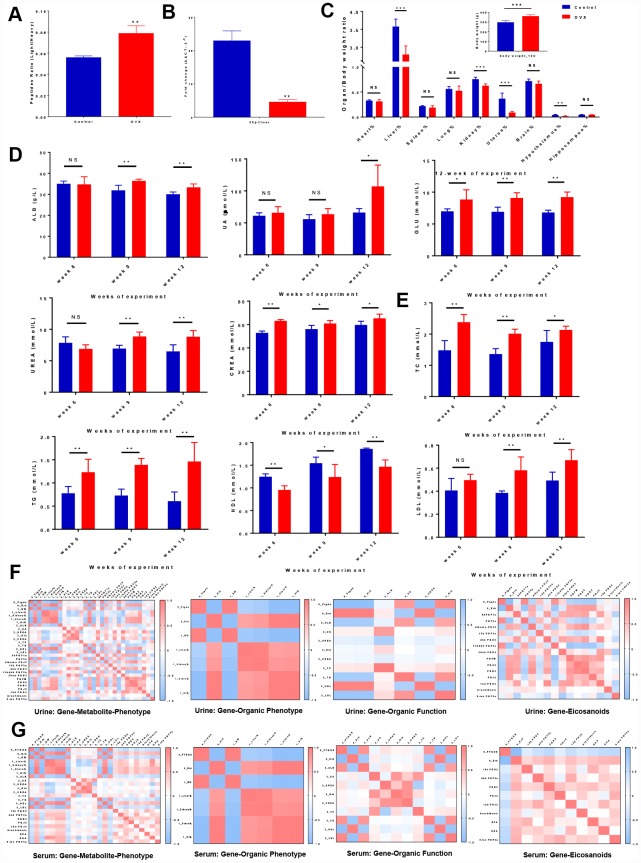
**Correlation analyses illustrated the relationships among ‘genes-metabolites-phenotypes’ in ER-depletion-induced renal lipid metabolism disorder.** (**A**) Relative peptide quantification in control and OVX rat urine samples. n=3, mean ± s.e.m.; (**B**) Real time PCR assays of uterus ERβ among control, OVX and OVX+E2 rats. n=3, mean ± s.e.m.; (**C**) Average weekly body weight (g) from 0 to 12 weeks before and after surgery and average organ/body weight ratio (organ %) at 12 weeks after surgery. (**D**) Kidney biochemical profiles in the sera of control and OVX rats. (**E**) Lipid biochemical profiles in the sera of control and OVX rats. n=6, mean ± s.d., compared to control rats, *p ˂ 0.05, **p ˂ 0.005, ***p ˂ 0.0005. Correlation heat map representation of the differentially expressed gene and metabolite markers in organs, renal biochemistry, and lipid biochemistry phenotypes including genes clustered into organ phenotypes, subsets of genes clustered into organ functions and subsets of genes clustered into eicosanoid markers in urine (**F**) and serum (**G**).

Ptgds is a clinical diagnostic marker that is expressed on the extracellular membranes of tissues and fluctuates in body fluids under various pathological conditions; in addition, this marker is essential for the generation of various prostanoids with numerous physiological and pathophysiological functions. We performed MRM-based targeted proteomics analysis by measuring the ratio of heavy-isotope-labelled peptides in urine samples by LC-MS in MRM mode, we observed that the Ptgds levels were significantly higher in the OVX rats than in the control rats. (Figure 3A, Supplementary Table 12). Since Ptgds promoter activity was mainly modulated by ERβ, which is consistent with the high expression pattern observed for uterus ERβ in the cardiovascular, reproductive, and urinary systems (the brain, heart, ovaries, and kidneys). We performed real time polymerase chain reaction (PCR) analysis for ERβ assay (ref. NM_012754.1, 204 bp, 60°C, Servicebio: forward primer: 5′- CTGGGTGATTGCGAAGAGTGG -3′ and reverse primer: 5′- GAGGACTTGTACCCTCGAAG CG -3′) (Figure 3B, Supplementary Table 11).

The body weights were monitored weekly, and organic morphological changes were assessed at 3-month intervals. The average weekly body weights were significantly higher in the OVX rats compared to the control rats. In contrast, the OVX rats displayed a significant deterioration of their organ/body weight ratios (heart, liver, spleen, lung, kidney, uterus, brain, hippocampus, and hypothalamus), demonstrating that ovariectomy accelerates organic tissue deterioration (Figure 3C, Supplementary Table 12).

We next assessed whether reduced ovarian function in the OVX rats had an effect on the biochemical profiles (kidney profile and lipid profile). For the kidney profile (organic function), all OVX rats presented a higher levels of serum albumin (ALB), uric acid (UA), urea, glucose (GLU), and creatinine levels compare to control rats (Figure 3D, Supplementary Table 12). For the lipid profile, increasing trends for the levels of TC, TGs, and LDL were observed, while a decreasing trend was observed for the HDL level in the OVX group (Figure 3E, Supplementary Table 12). These results revealed a relationship between renal lipid metabolism dysfunction and ovarian failure induced ER-depletion.

For multiscale evaluation, we analysed each set of omics data individually and then combined the ‘diseases-genes-metabolites’ to generate a ‘big picture’. By integrating the power of networks and evidence-based biological knowledge, the selected biomarkers (i.e., genes, metabolites, and phenotypes) were co-projected onto the networks to reveal important links among ovarian failure, ER-depletion, renal lipid metabolism, and other organs` diseases. We investigated whether strong Ptgds inhibition had a systematic effect on the body phenotypes (body weight and organic ratios) and organic functions (biochemistry parameters). ‘Gene-to-Gene’ correlation analysis showed a highly significant negative correlation between uterine ERβ and urine Ptgds, indicating that renal Ptgds overexpression caused by ovarian failure is mainly regulated by ERβ. We investigated whether strong Ptgds inhibition induced by ER-depletion had a systematic effect on the body phenotypes (body weight and organic ratios) and organic functions (biochemistry parameters). Accordingly, ‘Gene to Organic Phenotypes’ and ‘Gene to Organic Function’ correlation analyses showed that ERβ depletion was highly correlated with kidney and uterus organic phenotypes (kidney % coef.: 0.804, uterus % coef.: 0.999). Notably, urine Ptgds was highly correlated with kidney function (kidney % coef.: -0.804 and ALB coef.: 0.817) and lipid metabolism (body weight coef.: 0.979, TG coef.: 0.820, HDL coef.: -0.965, and LDL coef.: 0.921). However, uterus ERβ had the opposite correlation with kidney dysfunction and lipid metabolism disorder compared with those of urine Ptgds. (Figure 3F, 3G, Supplementary Table 13).

We further investigated whether the fluctuation of eicosanoid dynamics was due to Ptgds overexpression that suffered from ER-depletion. Thus, we next performed a correlation analysis on the ‘Gene to Eicosanoids’ relationship and showed that ERβ depletion was highly correlated with the Ptgds-catalysed urinary PGF_2a_ (coef.: 0.889), 11bdhk PGF_2a_ (coef.: 0.858), PGJ_2_ (coef.: -0.768), dihomo PGJ_2_ (coef.: -0.654), PGA_2_ (coef.: -0.738), PGB_2_ (coef.: -0.782), serum PGB_2_ (coef.: -0.602), and 15d PGJ_2_ (coef.: -0.503) levels.. In addition, PGJ_2_, 15d PGJ_2_, and dihomo PGJ_2_ were produced from PGD_2_ in the arachidonic acid pathway [26, 27]. These PGD_2_ derivates exhibit the opposite effects on the glucose homeostasis and adipocyte differentiation involved in kidney diseases; therefore, the relationship between the diminished amount of PGD_2_ derivates in bio-fluids and the overexpressed tissue-specific Ptgds indicated that these PGD_2_ derivates elicit their lipid toxicity stimulated by accumulating in extracellular space (Figure 3F, 3G, Supplementary Table 13). These data indicate that the ovarian failure induced accelerated degradation of ERβ in rats cause Ptgds overexpression in the kidney, and possibly altered the systemic lipid metabolism disorder resulting in HUK axis degenerative changes.

### Immunofluorescence (IF), Western blot (WB), real time polymerase chain reaction (PCR) assays and Spatial learning behavior test confirmed that ER-depletion reduce HUK functions attribute to ERβ/Ptgds signalling pathway disturbance

**Figure 4:**
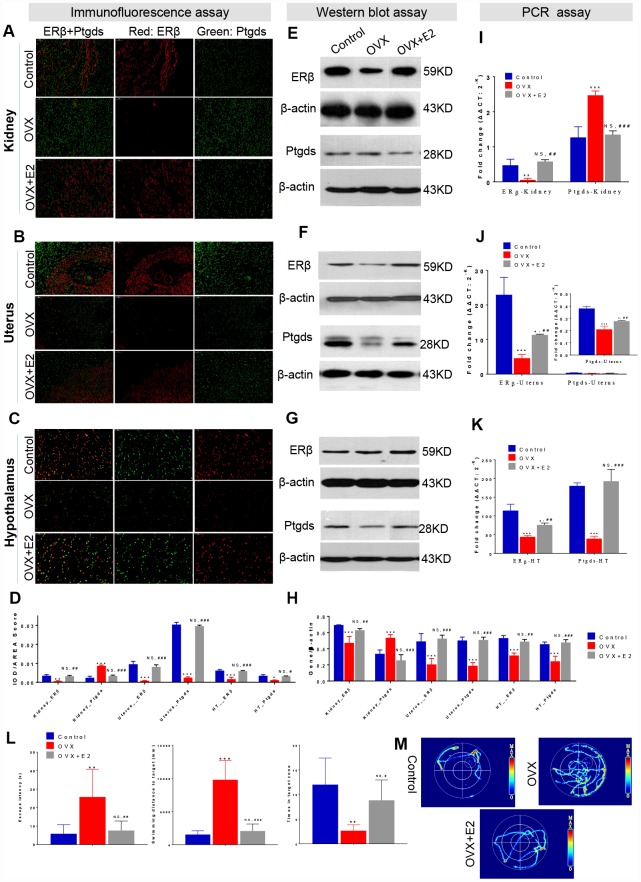
**ER-depletion reduce HUK functions attribute to ERβ/Ptgds signalling pathway disturbance.** Immunofluorescence (IF) analysis including the staining images (**A**–**C**) and AOD calculation data (**D**) in the left panel shows the double staining of ERβ (red) and Ptgds (green) from kidney (**A**), uterine (**B**), and hypothalamic (**C**) among control, OVX, and E2+OVX rats. Scale bar, 50 μM. The AOD ratio indicates the ratio of optical density (IOD) and values to staining area (AREA). Western blot (WB) analysis including the gel images (**E**–**F**) and protein loading data (**H**) in the middle panel present the ERβ and Ptgds proteins expression from kidney (**E**), uterine (**F**), and hypothalamic (**G**) among control, OVX, and E2+OVX rats. n=3, mean ± s.e.m. Real time PCR analysis of the transcription levels of ERβ and Ptgds expression various among control, OVX and OVX+E2 rats along ‘hypothalamus-uterus-kidney axis’ in the right panel (**I**–**K**). n=3, mean ± s.e.m., NS, not significant. Morris water maze test including location test and spatial learning test for control, OVX, and E2+OVX rats. As well as (**L**) escape latency duration, swimming length to the escape platform, number of crossings escape platform position, and (**M**) the images of total movement towards to the target for control, OVX, and E2+OVX rats. n=6, mean ± s.d., *p < 0.05, **p < 0.005, ***p < 0.0005 versus control rats; #p < 0.05, ##p < 0.005, ###p < 0.0005 *versus* control rats, NS, not significant.

To further confirm how the ER-depletion disturbing ERβ/Ptgds signalling pathway and reducing HUK function, a subset of OVX rats were administered E2 therapy (Figure 1, Study design), and IF, WB, PCR assays and spatial learning behavior test (Morris water maze test) were performed. In the IF analysis, double staining was conducted that red colour present ERβ and green colour present Ptgds. The AOD ratio indicates the ratio of optical density (IOD) and values to staining area (AREA), that a larger AOD ratio represents a higher protein expression level. As expected, ERβ was specifically localized in nucleus, Ptgds localized in cellular membrane or extracellular space. Compared to those of the control rats, OVX rats showed a weak ERβ signal in kidney, uterus and hypothalamus. Kidney from OVX rats showed strong Ptgds signal in tubules but had a dimished signal in uterus and hypothalamus (Figure 4A–4C). E2 therapy significantly restored the ERβ/Ptgds staining AOD scores in hypothalamus, uterus, and kidney (both the overexpressed protein and the control) (Figure 4D, Supplementary Table 14).

We next assessed the effects of ER-mediated marker genes as well as the antagonism of kidney function by WB assays. ERβ expression was significantly decreased in the kidney, uterus and hypothalamus of the OVX rats. More importantly, a sharp increase in Ptgds expression was observed in OVX rat kidneys compared to the control rats; however, decreased Ptgds expression was identified in the uterus and hypothalamus (Figure 4E–4G). All the parameters were rescued by E2 treatment (Figure 4H, Supplementary Table 14). In agreement with these results, PCR analysis for expression comparison in the same tissues indicated a significant decline in the mRNA level of ERβ in the kidneys, uterus, and hypothalamus after ovarian failure that had been rescued by E2 therapy (Figure 4I–4K, Supplementary Table 14). Ptgds expression in the kidneys was increased in OVX rats but was restored by E2 treatment (Figure 4I). Specifically, ovarian failure reduced the relative expression of uterus and hypothalamus Ptgds, while E2 therapy completely reversed the changes (Figure 4J, 4K). In a location test of Morris water maze test, which measures spatial learning activity that were associated with hypothalamus function, OVX rats showed decreased learning and recognize activities (increased escape latency duration and swimming length) compared to that of control rats. While E2 treatment rats generally spent less time to search the escape platform and reduced distance of travel than did OVX rats. There were no significant differences between the groups of control and E2 treatment rats (Figure 4L, Supplementary Table 14). In addition, in a spatial learning test of Morris water maze test, OVX rats exhibited a decline memory activity (reduced number of crossings escape platform position) than were control rats. No difference between control rats and E2 treatment rats was observed (Figure 4M, Supplementary Table 14).

Overall, E2 restoration effect indicated the relationship of ERβ/Ptgds signaling pathway. These data further confirmed that the upstream ERβ depletion activated renal Ptgds overexpression resulting renal lipid metabolism imbalance, decreased Ptgds transportation to hypothalamus and possibly continuing accelerate HUK function degeneration.

## DISCUSSION

In the past decade, scientific reports have shown that urinary Ptgds contributes to renal failure progression. Moreover, ERβ has been reported to stimulate Ptgds expression in female rat hearts; in addition, the engagement of the ER on Ptgds estrogen response elements (EREs) is essential for the acquisition of effector function, and the duration and strength of acute and chronic estrogen responses on Ptgds EREs are related to the integration of co-receptor signals [28]. As a member of the lipocalin family, Ptgds catalyses PGH_2_ isomerization into PGD_2_ and transports small hydrophobic molecules to the extracellular space and to various body fluids [29, 30]. Then, PGD_2_ is sequentially transformed into PGJ_2_ and into 15 deoxy PG^Δ12, 14^ J_2_ (15dPGJ_2_) [31]. The activation of Ptgds can affect lipid metabolic shifts, such as those of arachidonic acid [32], α-linolenic acid (ALA), and eicosanoid metabolism within the cyclooxygenase (COX) pathway [33, 34]. Ptgds secreted in urine is synthesized in Henle’s loop and the glomeruli and is mainly degraded by proteolysis after filtration from glomerular capillaries; then, its N-terminal-truncated form is ultimately excreted in urine [35]. Due to its low molecular weight and anionic properties, Ptgds can more easily pass the renal glomerular capillary wall than can serum albumin and can more accurately reflect changes in glomerular permeability. Indeed, lipid metabolism deficiency has been investigated in lipocalin-type PGD_2_ synthase (L-Pgds)-knockout (KO) mice and kidney dysfunction patients, which have a susceptibility to glucose intolerance, accelerated insulin resistance, and aggravated obesity [36, 37]. Although considerable research has explored the role of prostaglandins in the kidney, the main focus of such studies has been on the cardiovascular and insulin functions rather than on the influence of estrogen.

**Figure 5:**
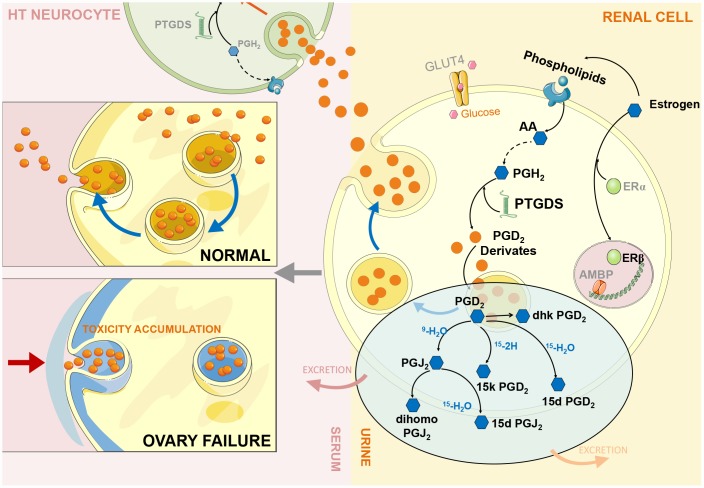
**ERβ/Ptgds signalling pathway imbalance disrupting renal lipid metabolism and continues influencing ‘hypothalamus-uterus-kidney axis’ (HUK axis) function.** During ovary failure, a significant downregulation of ERβ (green bubble) leads to PTGDS overexpression (green helix shape) due to persistent releasing ‘estrogen secretion’ instruction from hypothalamus after ovary failure. We call this ‘fake – immunity’. This would further activate the generation of PGD_2_ and its derivatives (orange bubbles, each compound represent by blue hexagon shape) in renal cells (yellow cell structure), and inhibit them exert into body fluid circulation through the autocrine and paracrine pathways to reach other intracellular targets (serum: light pink colour; urine: light yellow colour). Therefore, extensively accumulation of these Ptgds regulated metabolites promote kidney lipid toxicity progression (cytoskeleton destruction, grey blue colour part in left panel), then kidney microenvironments disturbance occurs; consequently, inhibition of Ptgds transmission along the ‘hypothalamus-uterus-kidney axis’ result in its decreased expression in hypothalamus neurocyte (green cell structure). The artwork material from SMART SERVIER MEDICAL ART (Free download, https://smart.servier.com).

Based on the above experimental data, we found a negative feedback mechanism in the HUK axis. When the ovary cannot secrete estrogen because of functional failure, the brain still orders the reproductive system to secrete hormones, thus causing the abnormal overexpression of estrogen-regulated renal genes, such as Ptgds, which we call a ‘fake’ response. In theory, Ptgds overexpression will increase the production of downstream metabolites that should have passed through the cell membrane after production, which will then distribute to the corresponding target organs through body fluid circulation. However, we observed an obvious decreasing trend for Ptgds-regulated metabolites in serum and an increasing trend in urine, indicating that these metabolites cannot enter the cardiovascular system through intercellular transfer and that the cell membrane lost its delivery function (Figure 5).

We further revealed that estrogen-regulated renal gene overexpression was responsible for the inhibition of the transfer microenvironment, which is an important component of intercellular signalling. Published studies have indicated that the transfer microenvironment refers to a lipid microenvironment composed of membrane-secreting proteins (exosomes), phospholipids, and glycolipids, and its most important function is to facilitate cell signal transduction through corresponding receptors and kinases. In the experiment, we observed a significant decrease in the AA levels in body fluids, indicating that the synthesis of AAs into phospholipids was obstructed, which disturbed the lipid microenvironment. The downstream metabolites of AA were also decreased to balance homeostasis by secreting into specific targets. The perturbation of the lipid microenvironment causes Ptgds-regulated downstream metabolites to excessively accumulate in cells or on cell membranes, which not only produces renal lipid toxicity but also destroys the original transmission function of the cell membrane; therefore, metabolites cannot be transferred between cells, which are why metabolites cannot reach target organs through the circulatory system. More importantly, this finding indicates that Ptgds accumulation in the kidney prevents Ptgds from entering the CNS through an autocrine or bypass secretion pathway where it exerts anti-inflammatory immunity, which may result in degenerative symptoms, such as Alzheimer's disease and sleeping disorders. We confirmed this inference by measuring the reduced Ptgds expression in the hypothalamus and decline spatial learning behaviour.

We found that abnormal GLU, TC, and TG levels were driven by extensive lipid metabolism disorders, such as hyperglycaemia, hyperlipaemia, and abdominal obesity. More importantly, many prostaglandins produced by Ptgds, such as PGD_2_ and 15d-PGJ_2_, were diminished, thus reducing their tumour-inhibiting activity, which may induce breast cancer. Although this pathological phenotype was not observed in this experiment, many published documents have confirmed such mechanisms.

## CONCLUSIONS

In conclusion, we present a comprehensive proteomic and metabolomic description of ER-depletion triggered renal function imbalances resulting HUK functions impairment at multiple levels. Our studies found that ERβ-regulated Ptgds overexpression has a significant effect on renal lipid metabolic disorders caused by ovarian failure and further affects brain function. Additional studies are needed to prove whether the dynamic monitoring of abnormal Ptgds expression and the resultant metabolic disorders are reproducible in humans, which may have greater potential for climacteric syndrome predication and therapeutic evaluation as an effective methodological strategy.

## MATERIALS AND METHODS

Detailed experimental procedures are provided in Supplementary files.

### Chemicals, antibodies, oligonucleotides and reagents

Optima LC grade acetonitrile (ACN) were purchased from Merck, formic acid were purchased from Fluka, water were obtained from Watson. Primary antibodies were purchased from Abcam: ERβ (Abcam, ab3576, RRID: AB_303921), Ptgds (Abcam, ab182141, RRID: AB_2783784),

### Animals

SPF female Sprague - Dawley rats (6 - 8 weeks) were obtained from the Fourth Military Medical University Animal Centre. For further details, see Supplementary Experimental Procedures.

### Sample preparation

### Sample preparation for the urinary proteome

For further details, see Supplementary Experimental Procedures.

### Sample preparation for the urinary and serum metabolome

For further details, see Supplementary Experimental Procedures.

### Urinary proteome analysis

### SDS-PAGE processing

For further details, see Supplementary Experimental Procedures.

### iTRAQ labelling and 2D-HPLC-MS/MS analysis

For further details, see Supplementary Experimental Procedures.

### Urinary proteomic data processing

For further details, see Supplementary Experimental Procedures.

### Gene ontology (GO) annotation and network analyses

For further details, see Supplementary Experimental Procedures.

### LC-MS MRM for eicosanoid metabolite determination

For further details, see Supplementary Experimental Procedures.

### Metabolite biomarker selection and pathway enrichment

For further details, see Supplementary Experimental Procedures.

### Phenotypes evaluation

For further details, see Supplementary Experimental Procedures.

### LC-MS scheduled multiple reaction monitoring (MRM) analysis for targeted protein determination

For further details, see Supplementary Experimental Procedures.

### Targeted protein confirmation by Immunofluorescence (IF), Western blot (WB), real time PCR and Spatial learning behavior test

For further details, see Supplementary Experimental Procedures.

### Statistical analysis

GraphPad Prism 7.0 software (GraphPad Software, La Jolla, CA, USA) was used for the statistical analysis. The statistical analysis for two groups comparisons were conducted using a two-tailed unpaired Student’s t-test, for three groups were conducted using one-way ANOVA, followed by Sidak`s multiple comparisons test. Differences with *p* values ˂ 0.05 were considered significant. No statistical methods were used to predetermine the sample size.

## Supplementary Material

Supplementary Materials

Supplementary Figures

Supplementary Tables

Supplementary Table 1

Supplementary Table 3

Supplementary Table 13
