# Supplementary material for: ER-depletion lowering the 'hypothalamus-uterus-kidney' axis functions by perturbing the renal ERβ/Ptgds signalling pathway
Source: Aging (Albany NY). 2019 Nov 10;11(21):9500–29. doi: 10.18632/aging.102401 (PMC6874469; doi:10.18632/aging.102401)
Supplement: Supplementary Materials [file aging-11-102401-s004.pdf]

SUPPLEMENTARY MATERIALS

SUPPLEMENTARY EXPERIMENTAL PROCEDURES

Chemicals, antibodies, oligonucleotides and reagents

Optima LC grade acetonitrile (ACN) were purchased from Merck, formic acid were purchased from Fluka, water were obtained from Watson. Primary antibodies were purchased from Abcam: ERβ (Abcam, ab3576, RRID: AB_303921), Ptgds (Abcam, ab182141, RRID:AB_2783784).

Animals

SPF female Sprague - Dawley rats (6 - 8 weeks) were obtained from the Fourth Military Medical University Animal Centre, maintained in metabolic cages under a 12-h light/dark cycle at 23 ± 2 °C and 55 ± 10% relative humidity and acclimatized for 5 days. Food and drinking water were provided regularly and unrestricted. Body weights were recorded. Rats were ovariectomized at 10 weeks of ages, OVX technical details for all experiments are available in previous study [1, 2]. The rats performed sham surgery was set as control group. For confirmed the targeted proteins, another 3-month Estradiol (E_2) therapy group (OVX+E_2, ig, E2 at 1.5 mg/kg/week) was considered. Animal handling and experimentation were carried in accordance with the guidelines of the Guide for the Care and Use of Laboratory Animals and were approved by the Laboratory Animal Care and Use Committee of the Shaanxi University of Chinese Medicine (Figure 1, Study design).

Sample preparation

Sample preparation for the urinary proteome

For proteomic analysis, 300-µL aliquots of urine were centrifuged twice at 4°C and 10,000×g for 20 min to separate urinary proteins from small molecules using an Amicon Ultra 0.5-mL Centrifugal Filter (10 kDa, UFC501096, Merck Millipore, Germany) according to the manufacturer's instructions. The proteins captured in the filter were washed with water three times at 4°C and 10,000×g for 30 min to remove possible interferences. Then, the concentrated ultrafiltrate was centrifuged at 3,000×g for 1 min and freeze-dried for protein determination. All urinary solutions were stored at -20°C until use.

Sample preparation for the urinary and serum metabolome

For metabolomic analysis, primary urinary and serum samples were prepared according published procedures [1].

Urinary proteome analysis

SDS-PAGE processing

The total protein concentration was measured in each sample using a BCA Protein Assay Kit (Thermo Scientific, Waltham, MA, USA) at an absorbance of 570 nm, referenced by standard curve. Urinary proteins were fractionated by sodium dodecyl sulfate - polyacrylamide gel electrophoresis (SDS - PAGE).

iTRAQ labelling and 2D-HPLC-MS/MS analysis

Each 200-µg extract sample was prepared following standard digestion, reduction, and alkylation procedures prior to isobaric tag for relative and absolute quantitation (iTRAQ) labelling [3]. Peptide labelling (114-117) with iTRAQ reagent was performed following the manufacturer's protocol. The four isotopic iTRAQ labelling samples were combined and were fractionated on a waters UPLC using a C18 column (waters BEH C18 2.1 ×50mm, 1.7um). Peptides were eluted at a flow rate of 600Ml / min with a linear gradient of 5~35% solvent B (acetonitrile) over 10 min, the solvent A is 20mM ammonium formate with pH adjusted to 10. The absorbance at 214 nm was monitored, and a total of 10 fractions were collected. The fraction was separated by nano-HPLC (Eksigent Technologies) on the secondary RP analytical column (Eksigent, C18, 3µm, 150 mm ×75µm), using a gradient mobile phase 98% acetonitrile - 2% milli Q water (A) and 0.1% formic acid (B), at a flow rate of 0.3 µL / min, the gradient program was as follows: 0-5 min: 5% B, 6-65 min: 5-40% B, 65-66 min: 40-80% B, 66-71 min: 80% B, 71-72 min: 80-5% B, and 72-90 min: 5% B. Full scan and tandem mass spectrometry experiments were duplicated again on an AB SCIEX TripleTOF 5600 system. Information-dependent data (IDA) acquisition mode was used to switch automatically between MS and MS/MS acquisition with the following parameters: an ion spray voltage of 2,500 V, curtain gas (CUR) of 25 p.s.i., a cycle time of 1 s, and a dwell time of 30 ms. MS spectra were acquired between 350~1250 m/z (molecular weight/valency) in high-resolution mode (40,000 resolution for full scans and 20,000 resolution for MS/MS scans) with rolling collision energy on via 250-ms accumulation time/spectrum and 50-mDa mass tolerance, and the 10 most intense precursors per cycle were selected for fragmentation with a dynamic exclusion time of 30 s. Data acquisition was conducted using Analyst 2.0 software (AB SCIEX).The collected samples were re-suspended in in 0.1% formic acid solution (0.1% formic acid - 2%

acetonitrile - 98% milli Q water) and analysed on a Eksigent C18 column (150 mm × 75 µm, 3 µm), using a gradient mobile phase 98% acetonitrile-2% milli Q water (A) and 0.1% formic acid (B), at a flow rate of 0.3 µL/min, the gradient program was as follows: 0-5 min: 5% B, 6-65 min: 5-40% B, 65-66 min: 40-80% B, 66-71 min: 80% B, 71-72 min: 80-5% B, and 72-90 min: 5% B.

Urinary proteomic data processing

Original data (*.wiff, *.wiff.scan) acquired from the MS/MS analysis in each LC run were converted to Mascot generic files (*.mgf) using ProteinPilot 4.5 software (AB SCIEX). The MS/MS peak lists of the formatted data were then searched by Mascot (Matrix Science, London, UK; version 2.3.02), spectra were searched against the UniProt database, and the taxonomy was set to Rat. Scaffold (version Scaffold_4.4.5, Proteome Software Inc., Portland, OR) was used to validate MS/MS based peptide and protein identifications.

Gene ontology (GO) annotation and network analyses

Protein annotation was subject to GO analysis on the basis of biological responses or disease analysis for biological processes, molecular functions, and cellular components. The differentially expressed proteome (> 1.5-fold changes in all ratios) from the iTRAQ LC-MS/MS analysis of urinary samples was interpreted using Ingenuity Pathway Analysis 9.1 (Ingenuity Systems, Mountain View, CA, USA, www.ingenuity.com) [4]. To conduct a pathway analysis and identify proteins connected to pathways of interest, both upregulated and downregulated proteins were computed in accordance with each network [5, 6].

We additionally used the hypothesis strategy GeneMANIA (application version 3.6.0, *Rattus norvegicus*, freely available at http://genemania.org) to generate hypotheses regarding gene function and calculate gene lists and weighting genes, the algorithm of which is designed to automatically weight networks on the basis of levance to the gene set. To evaluate the significance of selected proteins from the observed network, we performed multiple gene queries to identify the most closely connected genes among the networks and attributes selected from IPA enrichment. Physical interactions, pathways, and genetic interactions were generated, and the datasets relevant to ovarian failure and the pro-survival signalling network were collected [7–9].

LC-MS MRM for eicosanoid metabolite determination

The LC-MS semi-quantification analysis of eicosanoids was conducted on the AB QTrap 4500 - Agilent 1260 LC-ESI/MS system (AB SCIEX, Agilent Technologies). A 5-µL aliquot of each urine sample was injected onto an Agilent ZORBAX Eclipse Plus C_{18} column (100 mm × 4.6 mm, 5 µm; Agilent Technologies) with a mobile phase consisting of 0.1% formic acid in water (A) and 0.1% formic acid in acetonitrile (B). Gradient elution was carried out for 35 min at a flow rate of 0.8 mL/min, and the optimized gradient process was set as follows: 0-25 min: 0.2% -100% B, 25-30 min: 100% B, 30-31 min: 100%-0.2% B, and 31-35 min: 0.2% B. The column temperature was maintained at 30°C. Quantified MS data were obtained with an electrospray ionization (ESI) source in negative ion (NI, 4.5 kV) mode under the following conditions: CUR, 25 p.s.i.; temperatures, 450°C; gas source 1 and gas source 2, 40°C, respectively, referring to the method published by Yan Wang *et al.* [10]. All data were obtained using Analyst 1.6.2 software (Applied Biosystems), and the metabolites were quantified using Multiquant software (Applied Biosystems) (Supplementary Figure 1, and Supplementary Table 1).

Metabolite biomarker selection and pathway enrichment

To identify biomarkers and construct a metabolite set, a multivariate statistical analysis, including principal component analysis (PCA), partial least squares-discriminant analysis (PLS-DA), t-tests, and area under the curve (AUC) analysis, was applied using the MetaboAnalyst 4.0 online platform (available online at http://www.metaboanalyst.ca) [11]. For more consistent and robust biomarker selection and to reduce study biases to facilitate more robust biomarker identification across different experiments on ovarian failure, three batches of data with a sample size capacity of 85 were conducted using the biomarker 'meta-analysis' process on MetaboAnalyst 4.0 [12]. To reveal important links among 'genes, metabolites, and diseases', we next selected the network explorer using the SIMCA-P software (14.1 version) module to represent a comprehensive knowledge base of 'renal lipid metabolism disorder-genes-metabolites' induced by ovarian failure for interactive network visualization [13].

Phenotypes evaluation

Body weight was monitored weekly, while the organ to body weight ratio (including the liver, kidneys, spleen, uterus, cerebrum, and hypothalamus) were determined at 12 week of the experiment. Biochemical assays were evaluated 12 weeks after rats were OVX. Complete histopathology assays from organs were also performed.

LC-MS scheduled multiple reaction monitoring (MRM) analysis for targeted protein determination

To understand the underlying ovarian failure-driven transformation process in the 'hypothalamus-uterus-kidney axis', we performed MRM-based targeted

proteomics. Briefly, a representative peptide was chosedchosen for one targeted protein. Heavy isotope-labeled peptides were ordered from BANKPEPTIDE LTD.(Hefei China). Each sample was spiked in heavy isotope-labeled peptide. (the detailed optimization information for each protein is listed in Supplementary Table 2) [16, 17]. MRM experiments were performed on a 6500 QTRAP hybrid triple quadrupole/linear ion trap mass spectrometer (AB Sciex, Foster City, CA) interfaced with a Eksigent nano 1D plus system (Waters, Milford, MA). MRM transitions were monitored using unit resolution in both Q1 and Q3 quadrupoles to maximize specificity. Data analyses were performed using SKYLINE (version 2.6).

Targeted protein confirmation by Immunofluorescence (IF), Western blot (WB), real time PCR and Spatial learning behavior test

IF

For double staining with uterus, hypothalamus, and kidney tissues, primary antibody for ERβ (Abcam, ab3576, 1:100), or PTGDS (Abcam, ab182141, 1:3000) was incubated overnight at 4 °C . After the primary antibodies had been applied, the sections were sequentially incubated with HRP-conjugated secondary antibody (goat anti-rabbit IgG, ab6721, abcam) and CY3-conjugated secondary antibody (goat anti-rabbit IgG, ab6939, abcam). 4',6-diamidino-2-phenylindole (DAPI) was used for nuclear staining. The sections were observed under an upright fluorescence microscope (NIKON ECLIPSE C1, Nikon, Japan) connected to a scanning system (NIKON DS-U3, Nikon, Japan) coupled with the digital slide viewer NDP.scan (version 3.2.12, Hamamatsu Photonics, Shizuoka Pref., Japan). Image-Pro plus 6.0 software was used to measure the staining area to obtain the integrated optical density (IOD) and staining area (AREA) values. Then the AOD ratio indicates the ratio of IOD to AREA, that a larger AOD ratio represents a higher protein expression level.

WB

We next confirmed whether the selected anormaly expressed proteins had effect on ‘hypothalamus-uterus-kidney’ axis. The hypothalamus, uterus, and kidneys tissues were removed from control and OVX rats and subjected to western blot analysis. The proteins were transferred onto nitrocellulose membranes. After blocking in 5% BSA-TBST (Sigma), the membranes were incubated with the following primary antibodies from Abcam at 4°C overnight: ERβ (1:1000, ab3576), and Ptgds (1:1000, ab182141). Blots were then washed and incubated with the appropriate secondary antibody for 40 min at room temperature. Blot bands were visualized using chemiluminescence (ECL, WBKLS0500, Millipore). Relative band pixel intensities were semi-quantified using ImageJ software (NIH), and protein loading was normalized using an antibody against β-actin (A5441; Sigma-Aldrich) [15].

Real time PCR

100 mg of uterus, hypothalamus, and renal kidney were homogenized in TRIzol reagent (Servicebio) to extract total RNA, and 1 mg RNA was reverse-transcribed using RevertAid First Strand cDNA Synthesis Kit (#K1622, Thermo). Quantitative PCR was performed in a ViiaTM 7 Real-Time PCR System (Life Technologies, Grand Island, NY) using FastStart Universal SYBR Green Master (Rox) reagent (04 913 914 001, Roche). Assays were performed in triplicates and final values were normalized to ribosomal Ptgds or ERβ mRNA levels. The oligonucleotides (Invitrogen) for PCR as follows: rat Ptgds (ref. NM_013015.2, 296 bp, 60°C, Servicebio): forward primer: 5′- CAGCCCAACTTTCAACAAGACA -3′ and reverse primer: 5′- GCGTACTCATCGTAGTCG GTTTC -3′; rat ERβ (ref. NM_012754.1, 204 bp, 60°C, Servicebio): forward primer: 5`- CTGGGTGATTGCGA AGAGTGG -3′ and reverse primer: 5′- GAGGACTTGT ACCCTCGAAGCG -3′.

Spatial learning behavior test (Morris water maze test)

All behavioral testing protocols were approved by the Laboratory Animal Care and Use Committee of the Shaanxi University of Chinese Medicine. The Morris water maze (MWM) test for rats` location and spatial learning was conduct in a 130 cm diameter white plastic maze (130cm diameter ×50cm height) and surrounded by opacity curtains from 9:00–11:00 AM. The maze was filled with opaque water (Titanium powder dyed white, 20~22° C) and contained a goal (PVC circular escape platform: 10 cm diameter × 22cm height) submerged 1cm below the water surface to keep the rats out of sight.

Location test: After 5 days pretraining procedures, each rat (the head top was dyed in yellow colour) was placed into the water facing the wall on the randomized quadrant points, the timing is stared the moment that the rat is released. When the rat touched the platform and stayed for at least 3s, timing is stopped. Once the rats located the platform, it was allowed to stay for 10 s. Rats not finding the platform within 90s limit are either placed on the platform or guided to it. During each trial, the data of escape latency and swim length were monitored automatically from the digitized image *via* image tracking system (WMT-100S, Chengdu Technology & Market Corp., LTD, China).

Spatial learning test: The spatial learning test was conducted on continuous training day 6 after location test. The invisible platform was removed and the rat was released at a new start position and repeat the trial. During each trial, the data of total number of platform site crossings was monitored.

Statistical analysis

GraphPad Prism 7.0 software (GraphPad Software, La Jolla, CA, USA) was used for the statistical analysis. The statistical analysis for two groups comparisons were conducted using a two-tailed unpaired Student's t-test, for three groups were conducted using one-way ANOVA, followed by Sidak`s multiple comparisons test Differences with p values < 0.05 were considered significant. No statistical methods were used to predetermine the sample size.

REFERENCES

1. Liu YR, Huang RQ, Xiao BK, Yang JY, Dong JX. (1)H NMR metabolic profiling analysis offers evaluation of Nilestriol treatment in ovariectomised rats. Mol Cell Endocrinol. 2014; 387:19–34. https://doi.org/10.1016/j.mce.2014.02.007 PMID:24565896

2. Liu YR, Xiao BK, Yang JY, Guo CH, Shen SJ, Tang ZS, Dong JX, Huang RQ. 1 H-NMR and HPLC–MS/MS-based global/targeted metabolomic evaluation of Hypericum perforatum L. intervention for menopause. J Funct Foods. 2015; 17:722–41. https://doi.org/10.1016/j.jff.2015.06.023

3. Shevchenko A, Tomas H, Havlis J, Olsen JV, Mann M. In-gel digestion for mass spectrometric characterization of proteins and proteomes. Nat Protoc. 2006; 1:2856–60. https://doi.org/10.1038/nprot.2006.468 PMID:17406544

4. Ix JH, Katz R, Bansal N, Foster M, Weiner DE, Tracy R, Jotwani V, Hughes-Austin J, McKay D, Gabbai F, Hsu CY, Bostom A, Levey AS, Shlipak MG. Urine Fibrosis Markers and Risk of Allograft Failure in Kidney Transplant Recipients: A Case-Cohort Ancillary Study of the FAVORIT Trial. Am J Kidney Dis. 2017; 69:410–19. https://doi.org/10.1053/j.ajkd.2016.10.019 PMID:28024930

5. Ashburner M, Ball CA, Blake JA, Botstein D, Butler H, Cherry JM, Davis AP, Dolinski K, Dwight SS, Eppig JT, Harris MA, Hill DP, Issel-Tarver L, et al, and The Gene Ontology Consortium. Gene ontology: tool for the unification of biology. Nat Genet. 2000; 25:25–29. https://doi.org/10.1038/75556 PMID:10802651

6. Harris MA, Clark J, Ireland A, Lomax J, Ashburner M, Foulger R, Eilbeck K, Lewis S, Marshall B, Mungall C, Richter J, Rubin GM, Blake JA, et al, and Gene Ontology Consortium. The Gene Ontology (GO) database and informatics resource. Nucleic Acids Res. 2004; 32:D258–61. https://doi.org/10.1093/nar/gkh036 PMID:14681407

7. Franz M, Rodriguez H, Lopes C, Zuberi K, Montojo J, Bader GD, Morris Q. GeneMANIA update 2018. Nucleic Acids Res. 2018; 46:W60–W64. https://doi.org/10.1093/nar/gky311 PMID:29912392

8. Vlasblom J, Zuberi K, Rodriguez H, Arnold R, Gagarinova A, Deineko V, Kumar A, Leung E, Rizzolo K, Samanfar B, Chang L, Phanse S, Golshani A, et al. Novel function discovery with GeneMANIA: a new integrated resource for gene function prediction in Escherichia coli. Bioinformatics. 2015; 31:306–10. https://doi.org/10.1093/bioinformatics/btu671 PMID:25316676

9. Pirooznia M, Wang T, Avramopoulos D, Potash JB, Zandi PP, Goes FS. High-throughput sequencing of the synaptome in major depressive disorder. Mol Psychiatry. 2016; 21:650–55. https://doi.org/10.1038/mp.2015.98 PMID:26216301

10. Wang Y, Armando AM, Quehenberger O, Yan C, Dennis EA. Comprehensive ultra-performance liquid chromatographic separation and mass spectrometric analysis of eicosanoid metabolites in human samples. J Chromatogr A. 2014; 1359:60–69. https://doi.org/10.1016/j.chroma.2014.07.006 PMID:25074422

11. Xia J, Psychogios N, Young N, Wishart DS. MetaboAnalyst: a web server for metabolomic data analysis and interpretation. Nucleic Acids Res. 2009; 37:W652–60. https://doi.org/10.1093/nar/gkp356 PMID:19429898

12. Chong J, Soufan O, Li C, Caraus I, Li S, Bourque G, Wishart DS, Xia J. MetaboAnalyst 4.0: towards more transparent and integrative metabolomics analysis. Nucleic Acids Res. 2018; 46:W486–94. https://doi.org/10.1093/nar/gky310 PMID:29762782

13. Xia J, Gill EE, Hancock RE. NetworkAnalyst for statistical, visual and network-based meta-analysis of gene expression data. Nat Protoc. 2015; 10:823–44. https://doi.org/10.1038/nprot.2015.052 PMID:25950236

14. Yu J, Kane S, Wu J, Benedettini E, Li D, Reeves C, Innocenti G, Wetzel R, Crosby K, Becker A, Ferrante M, Cheung WC, Hong X, et al. Mutation-specific antibodies for the detection of EGFR mutations in non-small-cell lung cancer. Clin Cancer Res. 2009; 15:3023–3028.

https://doi.org/10.1158/1078-0432.CCR-08-2739 PMID:19366827

15. Mao K, Quipildor GF, Tabrizian T, Novaj A, Guan F, Walters RO, Delahaye F, Hubbard GB, Ikeno Y, Ejima K, Li P, Allison DB, Salimi-Moosavi H, et al. Late-life targeting of the IGF-1 receptor improves healthspan and lifespan in female mice. Nat Commun. 2018; 9:2394. https://doi.org/10.1038/s41467-018-04805-5 PMID:29921922

16. Chen C, Liu X, Zheng W, Zhang L, Yao J, Yang P. Screening of missing proteins in the human liver proteome by improved MRM-approach-based targeted proteomics. J Proteome Res. 2014; 13: 1969–78. https://doi.org/10.1021/pr4010986 PMID:24597967

17. Kim Y, Jeon J, Mejia S, Yao CQ, Ignatchenko V, Nyalwidhe JO, Gramolini AO, Lance RS, Troyer DA, Drake RR, Boutros PC, Semmes OJ, Kislinger T. Targeted proteomics identifies liquid-biopsy signatures for extracapsular prostate cancer. Nat Commun. 2016; 7:11906. https://doi.org/10.1038/ncomms11906 PMID:27350604
