# Supplementary material for: ER-depletion lowering the 'hypothalamus-uterus-kidney' axis functions by perturbing the renal ERβ/Ptgds signalling pathway
Source: Aging (Albany NY). 2019 Nov 10;11(21):9500–29. doi: 10.18632/aging.102401 (PMC6874469; doi:10.18632/aging.102401)
Supplement: Supplementary Figures [file aging-11-102401-s006.pdf]

SUPPLEMENTARY FIGURES

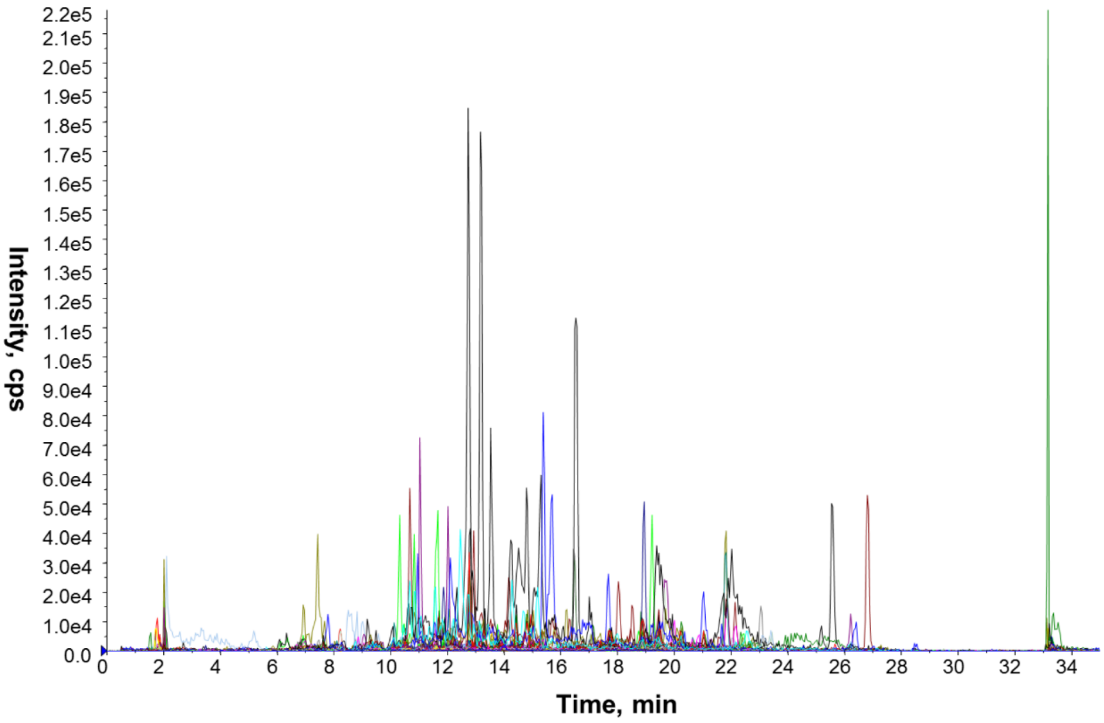

Supplementary Figure 1. Representative HPLC-ESI/MS MRM total ion chromatograms (TIC) of 155 eicosanoids.

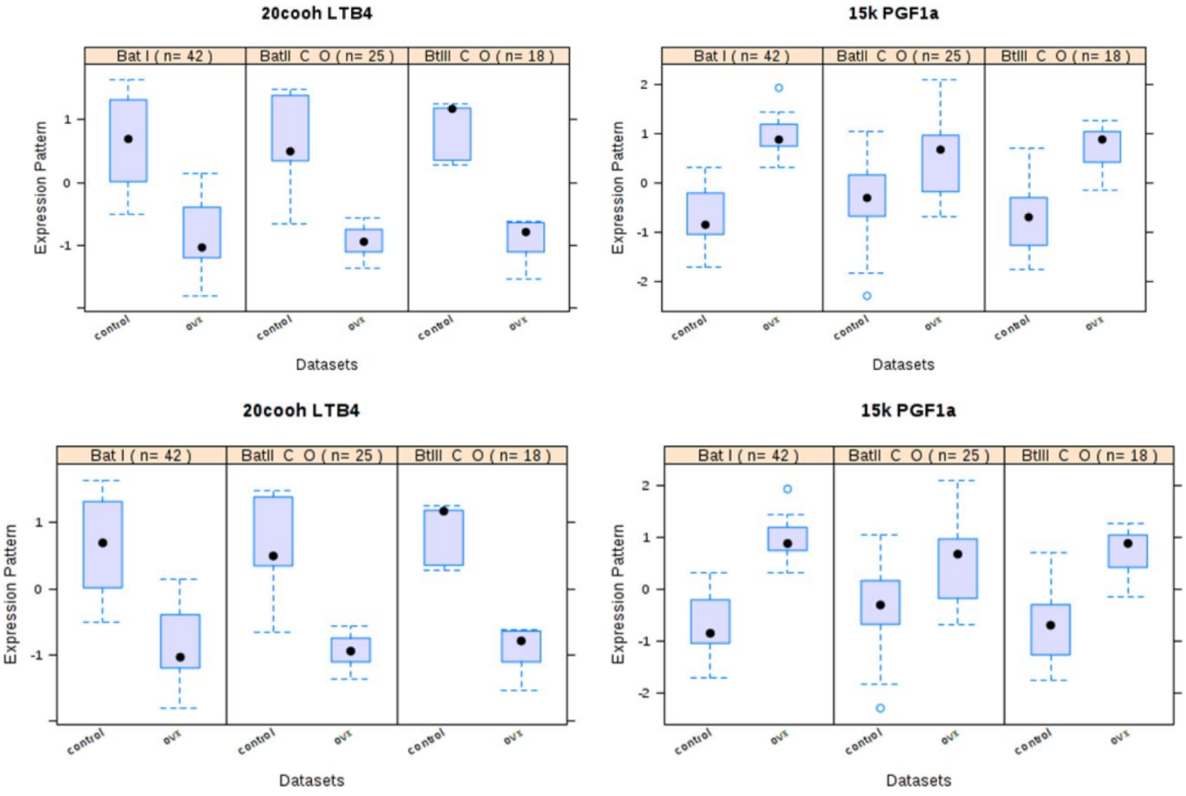

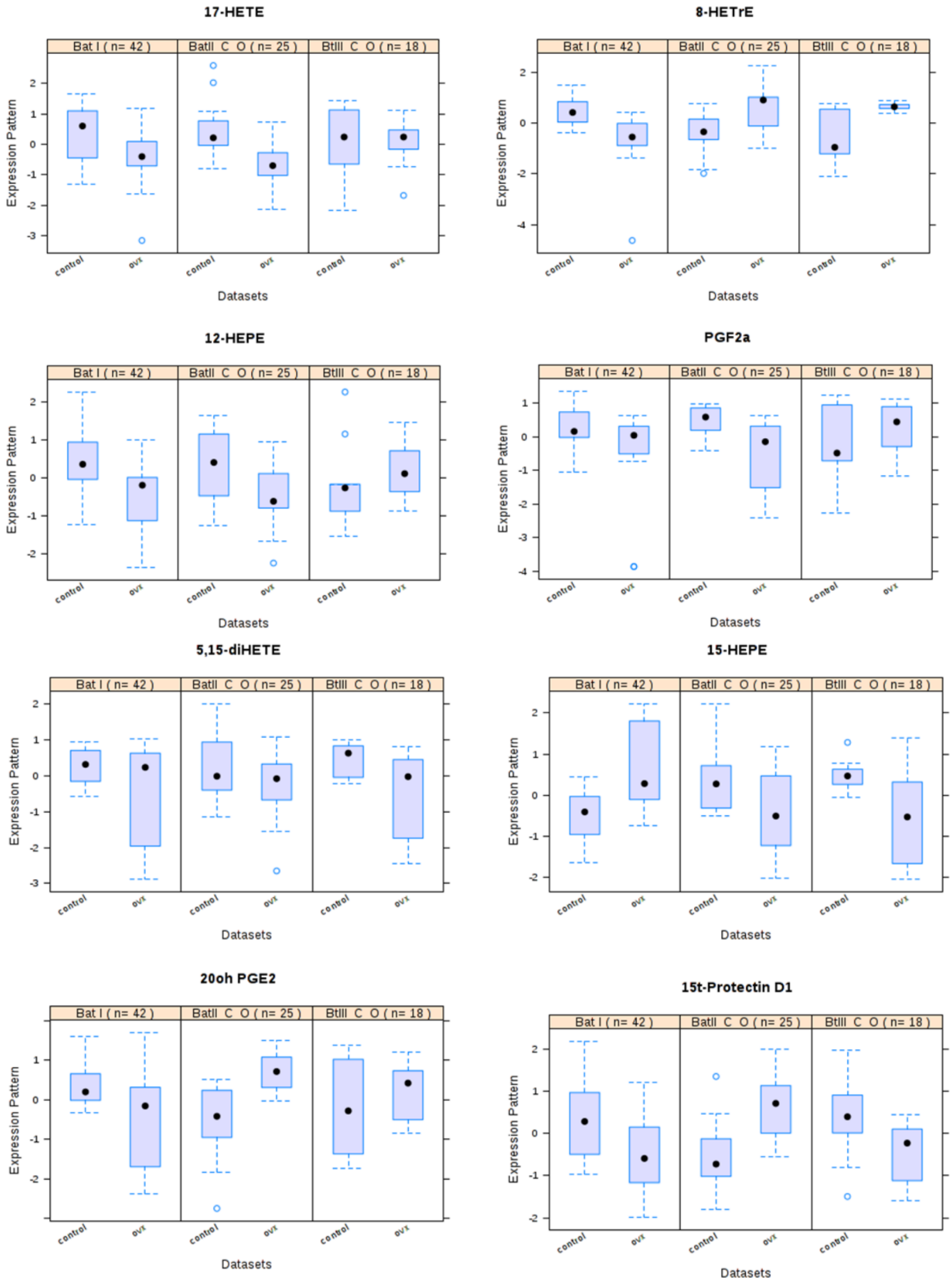
17-HETE
8-HETrE
12-HEPE
PGF2a
5,15-diHETE
15-HEPE
20oh PGE2
15t-Protectin D1
Bat I (n= 42)
BatII C O (n= 25)
BtIII C O (n= 18)
control
ovx
Expression Pattern
Datasets

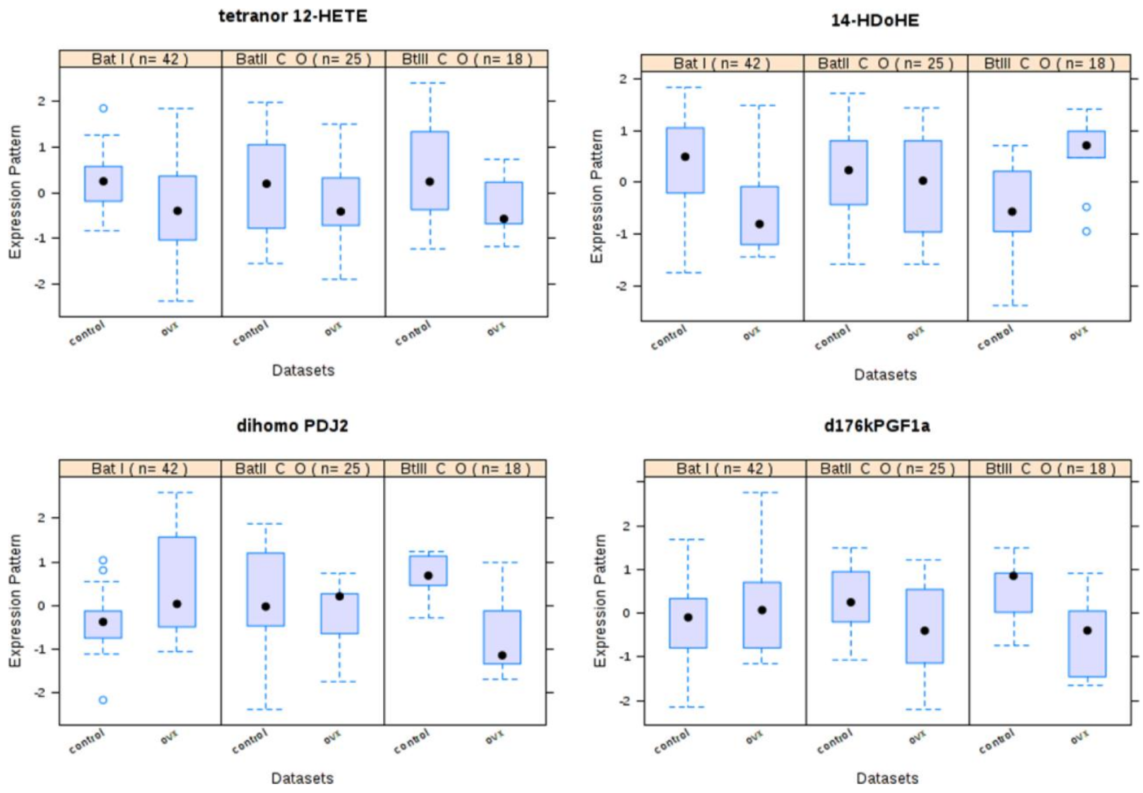

Supplementary Figure 2. Box plot of the expression pattern of the selected feature between the two experimental groups across all studies. The expression pattern is on the y-axis, and the group labels are on the x-axis. The median expression for the feature is indicated with a black dot in the center of the boxplot.
