# Supplementary material for: ER-depletion lowering the 'hypothalamus-uterus-kidney' axis functions by perturbing the renal ERβ/Ptgds signalling pathway
Source: Aging (Albany NY). 2019 Nov 10;11(21):9500–29. doi: 10.18632/aging.102401 (PMC6874469; doi:10.18632/aging.102401)
Supplement: Supplementary Tables [file aging-11-102401-s005.pdf]

SUPPLEMENTARY TABLES

Please browse Full Text version to see the data of Supplementary Table 1.

Supplementary Table 1. Optimized MRM pairs of LC/MS–MS for the 155 eicosanoids.

Supplementary Table 2. Targeted proteins MRM for Q1>Q3, DP, CE Optimization.

Protein	Peptide	Q1*	Q3*	DP	CE	ion type	Lable**
Prostaglandin-H2 D-isomerase	DQGLTEEDIVFLPQPDK	648.658072	844.4563	78.4	32.9	+3y7	light
		648.6581	697.3879	78.4	32.9	+3y6	light
		648.6581	584.3039	78.4	32.9	+3y5	light
		648.6581	301.1143	78.4	32.9	+3b3	light
		651.3295	852.4705	78.4	32.9	+3y7	heavy
		651.3295	705.4021	78.4	32.9	+3y6	heavy
		651.3295	592.3181	78.4	32.9	+3y5	heavy
		651.3295	301.1143	78.4	32.9	+3b3	heavy

*Q1 is the peptide precursor ion, and Q3 is the peptide product ion, DP is the declustering potential, and CE is the collision energy.
**Light: target peptide; Heavy: standards.

Please browse Full Text version to see the data of Supplementary Table 3.

Supplementary Table 3. Enrichment list of differentially expressed proteins on cellular component, cellular function, and biochemical process. (cut-off valure 1.5-fold change, p value < 0.05).

Supplementary Table 4. Top 5 disease and disorders predicted by IPA.

Disease	p-value	Molecules count	Molecules
Neurological Disease	3.61E-09-4.26E-03	22	DPP4,PVALB,C3,ACTB,CP,SOD1,PTGDS,GSN,LIFR,HSPA8,COL1A1,CDH1,CTSA,MYH2,ATP5B,GAA,ANXA1,EIF4A1,MYH3,PSAP,AFM,PLAU
Reproductive System Disease	1.23E-04-2.98E-03	20	DPP4,SERPINA6,MYH6,C3,LPO,CP,KRT5,PTGDS,SOD1,GSN,LIFR,COL1A1,CDH1,CAMP,WFDC2,ANXA1,EIF4A1,SLC3A1,AFM,PLAU
Metabolic Disease	1.52E-04-4.26E-03	17	DPP4,C3,ACTB,CP,PTGDS,SOD1,GSN,HSPA1L,COL1A1,Glycam1,CTSA,ANXA1,GAA,PSAP,AFM,PLAU,NPC2
Renal and Urological System Development and Function	1.56E-04-1.86E-03	4	CDH1,CAMP,C3,PLAU
Renal and Urological Disease	4.59E-04-2.13E-03	5	PVALB,DPP4,C3,PSAP,PTGDS

Supplementary Table 5. Top toxicity functions predicted by IPA.

Name	p-value	Overlap (select/identify)
Acute Renal Failure Panel (Rat)	9.18E-06	6.5 % (4/62)
Long-term Renal Injury Anti-oxidative Response Panel (Rat)	6.66E-04	11.1 % (2/18)
Positive Acute Phase Response Proteins	1.86E-03	6.7 % (2/30)
Persistent Renal Ischemia-Reperfusion Injury (Mouse)	1.86E-03	6.7 % (2/30)

Supplementary Table 6. Top 5 molecular and cellular functions predicted by IPA.

Name	p-value	Molecules count	Molecules
Cell Morphology	8.51E-08-3.31E-03	20	PVALB,DPP4,MYH6,C3,ACTB,AMBP,CP,PTGDS,SOD1,GSN,COL1A1,CTSA,CDH1,CAMP,MYH2,GAA,ANXA1,PSAP,PLAU,Tpm3
Tissue Morphology	8.51E-08-2.98E-03	18	MYH6,C3,AMBP,CP,PTGDS,SOD1,GSN,LIFR,COL1A1,CDH1,MYH2,CAMP,GAA,ANXA1,PSAP,PLAU,Tpm3,NPC2
Lipid Metabolism	4.41E-06-4.09E-03	15	DPP4,SERPINA6,PVALB,C3,CP,PTGDS,SOD1,GSN,HSPA1L,HSPA8,CAMP,ANXA1,PSAP,PLAU,NPC2
Cell-To-Cell Signaling and Interaction	5.27E-06-3.46E-03	16	DPP4,C3,ACTB,CD300LD,CP,SOD1,GSN,HSPA1L,LIFR,HSPA8,Glycam1,CDH1,CAMP,ANXA1,PSAP,PLAU
Cellular Function and Maintenance	5.27E-06-4.2E-03	17	S100vp,C3,ACTB,CP,SOD1,PTGDS,GSN,HSPA1L,HSPA8,COL1A1,CDH1,MYH2,CAMP,ANXA1,GAA,PSAP,PLAU

Supplementary Table 7. Top 5 associated network predicted by IPA.

ID	Top diseases and functions	Score	Focus molecules	Molecules in network
1	Cellular Movement, Hematological System Development and Function, Immune Cell Trafficking	42	18	AMBP, ANXA1, C3, CAMP, CDH1, COL1A1, Collagen type I, Collagen(s), CP, DPP4, elastase, ERK1/2, Fibrin, Fibrinogen, Growth hormone, GSN, HDL, IL12 (complex), Integrin, LDL, LIFR, LOC299282, LPO, Mmp, NPC2, PLAU, Pld, Pro-inflammatory Cytokine, Prss1 (includes others), PTGDS, Secretase gamma, SERPINA6, STAT5a/b, trypsin, WFDC2
2	Cellular Growth and Proliferation, Hematopoiesis, Cell Death and Survival	30	14	AMBP, AR, C2, CHUK, CNN3, Cpla2, CTSA, FAS, FNDC5, Glycam1, GSN, HNF4A, HPGDS, ITIH3, K Channel, LIFR, MAPK14, MYH2, MYOD1, PNAD, PODXL, PTGDS, PVALB, Pzp, RPS27A, Scgb1b27 (includes others), Scgb2b27 (includes others), SCPEP1, SUSD2, TGFB1, TNFAIP2, TP53, Tpm3, Tpm4, WISP3
3	Cell-To-Cell Signaling and Interaction, Carbohydrate Metabolism, Lipid Metabolism	11	7	AMBP,CALB1,CAMP,CD55,CD300LD,CLCF1,Cpla2,CXCR2,ERK,estrogen receptor, Focal adhesion kinase, FSH, IL36B, IL36G, Insulin, Jnk, JUN/JUNB/JUND, K Channel, Kng1/Kng1l1, LBP, LOC500183, Mac, Mapk, miR-146a-5p, MUC2, NFkB (complex), NFKBIA, P2RX7, P38 MAPK, p85 (pik3r), Pkc(s), PLA2G6, PLC, PTGDS, SLC3A1, Vegf

Supplementary Table 8. Top analysis-ready molecules predicted by IPA (FC).

GeneName	Abbreviations	FC	p value
Protein AMBP	Ambp↑	1.787	2.415E-07
Prostaglandin-H2 D-isomerase	Ptgds↑	1.521	1.591E-05
Annexin A1	Anxa1↓	0.601	9.868E-05
Complement C3	C3↓	0.568	4.663E-05
Cathelicidin antimicrobial peptide	Camp↓	0.377	5.069E-04
Collagen alpha-1(I) chain	Col1a1↓	0.562	9.670E-06
Leukemia inhibitory factor receptor	Lifr↓	0.651	9.233E-06
Myosin-6	Myh6↓	0.613	4.583E-04

Symbol	Score	Functions	Links
Camp	0.947235	disruption of cells of other organism, disruption of cells of other organism involved in symbiotic interaction, killing of cells in other organism involved in symbiotic interaction, killing of cells of other organism	http://www.ncbi.nlm.nih.gov/sites/entrez?db=gene&cmd=search&term=316010
Myh6	0.677982	blood circulation, structural molecule activity	http://www.ncbi.nlm.nih.gov/sites/entrez?db=gene&cmd=search&term=29556
Esr2	0.665631	response to estradiol, response to estrogen, vagina development	http://www.ncbi.nlm.nih.gov/sites/entrez?db=gene&cmd=search&term=25149
Lifr	0.649731	growth factor binding, muscle cell apoptotic process, negative regulation of muscle cell apoptotic process, organ regeneration, regeneration, regulation of muscle cell apoptotic process	http://www.ncbi.nlm.nih.gov/sites/entrez?db=gene&cmd=search&term=81680
Col1a1	0.597373	cellular response to acid, cellular response to amino acid stimulus, collagen, collagen fibril organization, extracellular matrix, extracellular matrix organization, extracellular matrix part, extracellular matrix structural constituent, extracellular structure organization, fibrillar collagen, growth factor binding, platelet-derived growth factor binding, protein heterotrimerization, proteinaceous extracellular matrix, response to amino acid, response to corticosteroid, response to estradiol, response to estrogen, skin development, structural molecule activity, wound healing	http://www.ncbi.nlm.nih.gov/sites/entrez?db=gene&cmd=search&term=29393
Ptgds	0.577912	response to corticosteroid, response to glucocorticoid	http://www.ncbi.nlm.nih.gov/sites/entrez?db=gene&cmd=search&term=25526
Anxa1	0.577502	binding, bridging, enzyme inhibitor activity, lipid localization, lipid transport, protein binding, bridging, response to corticosteroid, response to estradiol, response to estrogen, response to glucocorticoid, skin development, structural molecule activity	http://www.ncbi.nlm.nih.gov/sites/entrez?db=gene&cmd=search&term=25380
C3	0.568421	acylglycerol metabolic process, blood coagulation, blood microparticle, coagulation, glycerolipid metabolic process, hemostasis, neutral lipid metabolic process, positive regulation of endocytosis, regulation of body fluid levels, response to corticosteroid, response to estradiol, response to estrogen, response to glucocorticoid, triglyceride metabolic process, wound healing	http://www.ncbi.nlm.nih.gov/sites/entrez?db=gene&cmd=search&term=24232
Ambp	0.538466	blood microparticle, enzyme inhibitor activity	http://www.ncbi.nlm.nih.gov/sites/entrez?db=gene&cmd=search&term=25377

Supplementary Table 9. Network explore of the significant eight genes.

Function	FDR	Genes in network	Genes in genome
response to estrogen	1.01E-05	8	248
extracellular matrix	1.01E-05	8	262
response to estradiol	1.01E-05	7	161
wound healing	1.35E-05	8	287
blood microparticle	1.35E-05	6	97
collagen	0.000131	4	26
collagen fibril organization	0.000399	4	35
regeneration	0.000403	6	184
structural molecule activity	0.000686	6	205
platelet-derived growth factor binding	0.000693	3	11
response to amino acid	0.000693	5	110
extracellular matrix part	0.000732	5	113
cellular response to amino acid stimulus	0.000767	4	47
triglyceride metabolic process	0.002934	4	67
proteinaceous extracellular matrix	0.002934	5	156
acylglycerol metabolic process	0.004191	4	75
neutral lipid metabolic process	0.004399	4	77
extracellular matrix structural constituent	0.004607	3	23
organ regeneration	0.008058	4	92
negative regulation of muscle cell apoptotic process	0.008552	3	29
growth factor binding	0.010201	4	100
skin development	0.011398	5	222
blood coagulation	0.013091	4	110
response to corticosteroid	0.013091	5	232
hemostasis	0.013515	4	112
coagulation	0.014445	4	115
regulation of body fluid levels	0.015354	5	246
blood circulation	0.016329	5	251
regulation of muscle cell apoptotic process	0.019955	3	43
lipid localization	0.024317	4	136
cellular response to acid	0.025657	4	139
muscle cell apoptotic process	0.026932	3	49
extracellular matrix organization	0.038657	4	157
extracellular structure organization	0.038657	4	158
protein binding, bridging	0.047688	3	61
lipid transport	0.053154	4	174
vagina development	0.055889	2	11
SMAD binding	0.060897	3	68
binding, bridging	0.063153	3	70
protein heterotrimerization	0.063153	2	12
killing of cells in other organism involved in symbiotic interaction	0.069923	2	13
disruption of cells of other organism involved in symbiotic interaction	0.069923	2	13
enzyme inhibitor activity	0.072136	4	197
positive regulation of endocytosis	0.086299	3	82

killing of cells of other organism	0.086299	2	15
glycerolipid metabolic process	0.086299	4	211
disruption of cells of other organism	0.086299	2	15
response to glucocorticoid	0.09548	4	218

Supplementary Table 10. Kyoto Encyclopedia of Genes and Genomes (KEGG) pathway enrichment analysis on metaboanalyst platform with 'Joint Pathway Analysis' mode.

Pathway name	Match status	p	-log(p)	Holm p	FDR	Impact
Arachidonic acid metabolism	2/82	0.00327	5.7229	0.69655	0.69655	0.05
Taurine and hypotaurine metabolism	1/9	0.009941	4.6111	1	0.93978	0.33333
Cardiac muscle contraction	1/12	0.013236	4.3248	1	0.93978	0.36364
Hypertrophic cardiomyopathy (HCM)	1/25	0.027411	3.5968	1	1	0.57143

Supplementary Table 11. Differentially expressed features by individual study and from meta-analysis.

	ID	BAT I	BAT II	BAT III	Combined Tstat	Combined Pval	Combined vip	Combined AUC
1	20cooh LTB4	-1.469	-1.866	-1.790	-83.112	1.220E-13	1.747	0.990
2	15-oxoETE	1.750	1.235	1.594	71.761	1.400E-11	1.390	0.911
3	15k PGF2a	1.703	-0.072	1.751	63.377	3.630E-10	1.412	0.981
4	LXB4	1.607	-0.457	1.575	53.991	1.940E-08	1.283	0.894
5	11bdhk PGF2a	1.402	-0.034	1.770	50.453	8.550E-08	1.436	0.971
6	6kPGF1a	1.556	0.232	-0.233	38.183	9.265E-06	1.658	0.829
7	5,15-diHETE	-0.665	-1.555	-1.084	-34.280	4.054E-05	1.366	0.924
8	8-iso PGF3a	-1.203	-1.169	-0.299	-33.891	4.620E-05	1.588	0.925
9	11-HEPE	1.030	1.546	-1.065	33.498	5.273E-05	1.230	0.843
10	PGB2	0.031	-1.828	-0.566	-33.413	5.273E-05	1.286	0.889
11	12-HEPE	-0.998	-1.434	0.162	-30.889	1.441E-04	1.487	0.940
12	PGF2a	0.806	1.515	0.289	29.857	2.059E-04	1.216	0.771
13	dihomo PGJ2	0.885	-1.527	-1.418	-29.465	2.294E-04	1.542	1.000
14	PGFM	1.006	1.388	-0.877	29.314	2.314E-04	1.348	0.870
15	Arachidonic	-0.837	1.784	-0.485	25.986	8.576E-04	1.015	0.869
16	15d PGD2	-0.640	-0.850	-1.204	-24.361	1.530E-03	1.376	0.905
17	2,3-dinor TXB2	-0.627	-1.263	-0.332	-21.719	3.817E-03	1.186	0.979
18	5,6-diHETrE	-0.517	-1.268	-0.443	-20.682	5.474E-03	1.374	0.918
19	PGA2	0.392	-1.618	0.006	-20.446	5.935E-03	1.000	0.750
20	20oh PGE2	-0.845	-0.243	-0.292	-16.965	0.020	1.172	0.854
21	dhk PGD2	-0.613	-0.240	-0.852	-16.264	0.025	1.310	0.866
22	PGJ2	0.521	0.670	-1.480	-14.545	0.045	1.613	0.750

(cut-off criterion: FC> 1.5 (OVX/con), p-value <0.05, AUC > 0.75, VIP > 1).

Supplementary Table 12. The phenotype, serum biochemistry analysis.

**Protein quantification (n=3, mean ± s.e.m)**	**Urine Ptgds_ Peptides Ratio (Light/Heavy)_MRM**					**Uterus ERβ Fold change (ΔΔCT: 2-K)**				
Control_12 week	0.056±0.001					22.96±2.909				
OVX_12 week	0.079±0.004**					4.61±0.618**				
**Organ/body weight ratio (%) (n=6, mean ± s.d.)**	**Body Weight (g)**	**Heart %**	**Liver%**	**Spleen%**	**Lung%**	**Kidney%**	**Uterus%**	**Brain%**	**Hypothalamus %**	**Hippocampus %**
Control_12 week	297.12±16.66	0.33±0.02	3.58±0.21	0.22±0.01	0.56±0.05	0.76±0.04	0.37±0.11	0.72±0.04	0.043±0.013	0.047±0.004
OVX_12 week	360.22±15.04***	0.31±0.03^{NS}	2.80±0.24***	0.19±0.04^{NS}	0.52±0.10^{NS}	0.623±0.04***	0.09±0.02***	0.66±0.05^{NS}	0.017±0.009**	0.047±0.002^{NS}
**Biochemical Index**	**Kidney function profiles**					**Lipid profiles**				
n=6, mean ± s.d.	ALB (g/L)	UA (μmol/L)	UREA (mmol/L)	GLU (mmol/L)	CREA (mmol/L)	TC (mmol/L)	TG (mmol/L)	HDL (mmol/L)	LDL (mmol/L)	
Control_6 week	35.00±1.279	61.21±4.453	7.86±0.951	6.99±0.384	52.87±1.537	1.48±0.310	0.78±0.145	1.25±0.060	0.41±0.104	
OVX_6 week	34.73±3.616	65.78±9.537	6.90±0.643	8.84±1.526*	63.13±1.102**	2.38±0.244**	1.23±0.279**	0.96±0.091**	0.50±0.051	
Control_9 week	31.87±2.420	55.87±6.897	6.94±0.532	6.92±0.728	56.13±3.105	1.36±0.171	0.73±0.140	1.55±0.133	0.39±0.018	
OVX_9 week	36.40±0.817**	63.62±8.757	8.87±0.695**	9.09±0.803**	60.9±2.578*	2.01±0.142**	1.39±0.140**	1.24±0.277*	0.58±0.116**	
Control_12 week	30.02±1.072	66.23±6.028	6.49±1.053	6.81±0.357	59.73±3.075	1.75±0.365	0.61±0.199	1.86±0.019	0.49±0.072	
OVX_12 week	33.32±1.606**	107.12±33.284*	8.83±0.972**	9.21±0.801**	65.33±3.554*	2.13±0.119*	1.47±0.409**	1.47±0.150**	0.67±0.091**	

*p < 0.05, **p < 0.005, ***p < 0.0005 versus control rats. NS, not significant.

Please browse Full Text version to see the data of Supplementary Table 13

Supplementary Table 13. 'Gene-Metabolite-Phenotype' network analysis (urine & serum).

Supplementary Table 14. Western blot, immunohistochemistry and peptide MRM quantification results.

	Kidney		Uterus		Hypothalamus	
	**ERβ**	**Ptgds**	**ERβ**	**Ptgds**	**ERβ**	**Ptgds**
IF IOD/AREA score						
Control	0.0035±0.0003	0.0024±0.0004	0.0095±0.0009	0.0304±0.0007	0.0062±0.0003	0.0034±0.0004
OVX	0.0006±0.0001**	0.0087±0.0002***	0.0008±0.0001***	0.0024±0.0003***	0.0017±0.0006***	0.0011±6.849E-05*
OVX+E2	0.0032±0.0002NS,##	0.0034±0.0002 NS,###	0.0081±0.0006 NS,###	0.0296±0.0003 NS,###	0.0059±0.0002 NS,###	0.0032±9.231E-05 NS,#
WB assay						
Control	0.693±0.002	0.336±0.028	0.490±0.056	0.502±0.024	0.532±0.018	0.455±0.016
OVX	0.473±0.046***	0.536±0.023***	0.206±0.043***	0.189±0.022***	0.316±0.018***	0.244±0.036***
OVX+E2	0.629±0.011 NS,##	0.256±0.040 NS,###	0.525±0.024 NS,###	0.509±0.019 NS,###	0.488±0.015 NS,##	0.477±0.020 NS,###
PCR assay						
Control	0.47±0.099	1.27±0.176	22.96±2.909	0.380±0.010	114.237±9.776	180.137±4.688
OVX	0.057±0.027**	2.47±0.071***	4.61±0.618***	0.210±0.015***	44.137±1.979***	39.210±3.761***
OVX+E2	0.58±0.030 NS,##	1.35±0.060 NS,###	9.31±2.040 *,##	0.227±0.003 *,##	76.173±2.864 *,##	192.753±18.308 NS,###
Morris water maze						
	Escape latency (s)		Swimming distance to target (mm)		Times in target zone	
Control	5.850±2.011		1530.117±233.608		12.000±2.206	
OVX	25.767±6.110**		9791.883±1187.346***		2.667±0.494**	
OVX+E2	7.600±2.099 NS,##		2062.400±430.285 NS,###		8.833±1.682 NS,#	

(n=3, mean ± SEM).

*p < 0.05, **p < 0.005, ***p < 0.0005 versus control rats; #p < 0.05, ##p < 0.005, ###p < 0.0005 versus OVX rats. NS, not significant.
