# Supplementary material for: ER-depletion lowering the 'hypothalamus-uterus-kidney' axis functions by perturbing the renal ERβ/Ptgds signalling pathway
Source: Aging (Albany NY). 2019 Nov 10;11(21):9500–29. doi: 10.18632/aging.102401 (PMC6874469; doi:10.18632/aging.102401)
Supplement: Supplementary Table 1 [file aging-11-102401-s002.docx]

**Supplementary Table 1.** Optimized MRM pairs of LC/MS–MS for the 155 eicosanoids.

| **NO.** | **Compound name** | **Retention time** | **Precursor/Product ions** | **DP** | **CE** | **CXP** |
| --- | --- | --- | --- | --- | --- | --- |
| 1 | 6kPGF1a | 10.81 | 369/245 | -60 | -34 | -13 |
| 2 | TxB2 | 1.97 | 369/169 | -50 | -22 | -13 |
| 3 | PGF2a | 33.46 | 353/193 | -50 | -23 | -13 |
| 4 | PGE2 | 7.57 | 351.1/271.1 | -50 | -23 | -13 |
| 5 | PGD2 | 7.7 | 351/271 | -50 | -23 | -13 |
| 6 | 11bPGF2a | 13.71 | 353/335 | -50 | -35 | -13 |
| 7 | TXB1 | 13.62 | 371/171 | -30 | -27 | -13 |
| 8 | PGF1a | 19.07 | 355/293 | -60 | -33 | -13 |
| 9 | PGE1 | 1.86 | 353.1/235 | -40 | -29 | -13 |
| 10 | PGD1 | 2.32 | 353/235 | -40 | -29 | -13 |
| 11 | d176kPGF1a | 11.72 | 367/163 | -60 | -40 | -13 |
| 12 | TXB3 | 33.46 | 367/169 | -40 | -27 | -13 |
| 13 | PGF3a | 1.9 | 351/193 | -50 | -30 | -13 |
| 14 | PGE3 | 8.63 | 349.1/269 | -30 | -24 | -13 |
| 15 | PGD3 | 8.66 | 349/269 | -30 | -24 | -13 |
| 16 | dihomo PGF2a | 15.18 | 381/337 | -40 | -37 | -13 |
| 17 | dihomo PGE2 | 13.33 | 379.1/299 | -40 | -37 | -13 |
| 18 | dihomo PGD2 | 13.4 | 379/299 | -40 | -37 | -13 |
| 19 | dihomo PDJ2 | 15.33 | 361.1/299 | -40 | -37 | -13 |
| 20 | dihomo 15dmPGD2 | 15.03 | 361/299 | -40 | -37 | -13 |
| 21 | 6k PGE2 | 6.73 | 367/331 | -40 | -25 | -13 |
| 22 | 6,15-dk-,dh-PGF1a | 10.32 | 369/267 | -40 | -37 | -13 |
| 23 | 15k PGF1a | 17.71 | 353.1/221 | -50 | -38 | -13 |
| 24 | 15k PGF2a | 3.01 | 351.1/219 | -50 | -32 | -13 |
| 25 | 15k PGE2 | 1.74 | 349.1/235 | -30 | -26 | -13 |
| 26 | 15k PGD2 | 10.88 | 349/235 | -30 | -40 | -13 |
| 27 | dh PGF2a | 10.75 | 355/283 | -60 | -29 | -13 |
| 28 | dhk PGF2a | 7.58 | 353/291 | -80 | -28 | -13 |
| 29 | dhk PGE2 | 6.71 | 351.1/207 | -40 | -26 | -13 |
| 30 | dhk PGD2 | 6.67 | 351/207 | -40 | -26 | -13 |
| 31 | bicyclo PGE2 | 1.68 | 333/175 | -40 | -30 | -13 |
| 32 | 11bdhk PGF2a | 17.7 | 353/221 | -60 | -37 | -13 |
| 33 | 9oh PGF2a | 6.34 | 369/192 | -60 | -35 | -13 |
| 34 | 20ohmPGF2a | 1.84 | 369/165 | -40 | -39 | -13 |
| 35 | 19oh PGE2 | 9.1 | 367/243 | -20 | -31 | -13 |
| 36 | 20oh PGE2 | 8.41 | 367/175 | -30 | -27 | -13 |
| 37 | 2,3-dinor 11b PGF2a | 1.91 | 325/227 | -30 | -22 | -13 |
| 38 | PGFM | 2.04 | 329/247 | -40 | -25 | -13 |
| 39 | PGEM | 14.99 | 327/291 | -30 | -23 | -13 |
| 40 | tetranor 12-HETE | 10.79 | 265/109 | -20 | -18 | -13 |
| 41 | 11bPGE2 | 7.59 | 351.1/271 | -40 | -23 | -13 |
| 42 | PGK2 | 23.4 | 349/249 | -40 | -31 | -13 |
| 43 | 12-HHTrE | 8.07 | 279/217 | -30 | -21 | -13 |
| 44 | 11-HETE | 4.05 | 319.1/167 | -40 | -23 | -13 |
| 45 | 11-HEPE | 1.71 | 317.1/215 | -40 | -20 | -13 |
| 46 | 13-HDoHE | 11.81 | 343/221 | -30 | -19 | -13 |
| 47 | PGA2 | 8.64 | 333.1/271 | -20 | -20 | -13 |
| 48 | PGB2 | 8.85 | 333/271.1 | -40 | -25 | -13 |
| 49 | 15d PGA2 | 7.61 | 315/255 | -40 | -20 | -13 |
| 50 | PGJ2 | 7.83 | 333/189 | -40 | -22 | -13 |
| 51 | 15d PGD2 | 9.26 | 333.2/271 | -30 | -22 | -13 |
| 52 | 15d PGJ2 | 8.74 | 315/203 | -30 | -20 | -13 |
| 53 | 5-iso PGF2aVI | 15.64 | 353/115 | -60 | -28 | -13 |
| 54 | 8-iso PGF2aIII | 9.09 | 353.1/193 | -40 | -33 | -13 |
| 55 | 9-HETE | 1.83 | 319/123 | -40 | -20 | -13 |
| 56 | 9-HEPE | 12.08 | 317/149 | -40 | -20 | -13 |
| 57 | 8-HDoHE | 1.74 | 343/109 | -40 | -20 | -13 |
| 58 | 16-HDoHE | 7.55 | 343/233 | -50 | -19 | -13 |
| 59 | 20-HDoHE | 8.9 | 343.1/241 | -30 | -18 | -13 |
| 60 | LTB4 | 1.84 | 335.1/195.1 | -45 | -23 | -13 |
| 61 | 20oh LTB4 | 10.44 | 351/195 | -40 | -23 | -13 |
| 62 | 20cooh LTB4 | 12.7 | 365/303 | -40 | -26 | -13 |
| 63 | 5,6-diHETE | 9.77 | 335/115 | -50 | -29 | -13 |
| 64 | 6t LTB4 | 1.2 | 335.1/195 | -45 | -22 | -13 |
| 65 | 12epi LTB4 | 1.79 | 335/195.1 | -45 | -22 | -13 |
| 66 | 6t,12epi LTB4 | 1.67 | 335.2/195 | -45 | -22 | -13 |
| 67 | 120xo LTB4 | 11.21 | 335/253 | -50 | -22 | -13 |
| 68 | LTC4 | 10.97 | 624.1/272.1 | -50 | -33 | -13 |
| 69 | LTD4 | 5.85 | 495.2/177 | -50 | -29 | -13 |
| 70 | LTE4 | 2.53 | 438.1/333 | -30 | -25 | -13 |
| 71 | 11t LTC4 | 2.02 | 624.1/272 | -50 | -34 | -13 |
| 72 | 11t LTD4 | 8.51 | 495.1/177 | -50 | -29 | -13 |
| 73 | 5-HETE | 15.5 | 319/115 | -40 | -20 | -13 |
| 74 | 5-HEPE | 2.07 | 317/115 | -30 | -22 | -13 |
| 75 | 7-HDoHE | 10.84 | 343/141 | -40 | -19 | -13 |
| 76 | 4-HDoHE | 7.51 | 343/101 | -60 | -18 | -13 |
| 77 | 9-HOTrE | 4.97 | 193/171 | -40 | -22 | -13 |
| 78 | 5-HETrE | 7.89 | 321/205 | -30 | -19 | -13 |
| 79 | 5,15-diHETE | 12.24 | 335/201 | -40 | -26 | -13 |
| 80 | 6R-LXA4 | 2.05 | 351/167 | -20 | -21 | -13 |
| 81 | 15R-LXA4 | 10.78 | 351/165 | -20 | -23 | -13 |
| 82 | LXB4 | 17.68 | 351/221 | -50 | -21 | -13 |
| 83 | Resolvin E1 | 11.51 | 349/195 | -40 | -20 | -13 |
| 84 | Resolvin D1 | 1.79 | 375/141 | -20 | -20 | -13 |
| 85 | Pretectin D1 | 9.15 | 359.1/153 | -20 | -20 | -13 |
| 86 | 15t-Protectin D1 | 8.68 | 359.2/153 | -20 | -27 | -13 |
| 87 | 10S-Protectin D1 | 9.15 | 359/153 | -20 | -21 | -13 |
| 88 | 8,15-diHETE | 9.18 | 335/235 | -40 | -26 | -13 |
| 89 | 15-HETE | 10.13 | 319.1/175 | -40 | -19 | -13 |
| 90 | 15-HEPE | 1.94 | 317/219 | -40 | -18 | -13 |
| 91 | 17 HDoHE | 12.39 | 343/229 | -20 | -19 | -13 |
| 92 | 13-HODE | 21.69 | 295.1/195 | -60 | -23 | -13 |
| 93 | 13-HOTrE | 1.79 | 293/195 | -40 | -28 | -13 |
| 94 | 13-HOTrE(y) | 19.14 | 293/193 | -40 | -19 | -13 |
| 95 | 15-HETrE | 19.95 | 321/221 | -30 | -21 | -13 |
| 96 | 8-HETE | 1.99 | 319.1/155 | -40 | -19 | -13 |
| 97 | 8-HEPE | 11.21 | 317/155 | -50 | -29 | -13 |
| 98 | 10-HDoHE | 9.87 | 343/153 | -50 | -19 | -13 |
| 99 | 8-HETrE | 10.02 | 321/157 | -20 | -22 | -13 |
| 100 | 14,15-LTC4 | 8.21 | 624/272 | -30 | -32 | -13 |
| 101 | 14,15-LTD4 | 8.46 | 495/177 | -60 | -25 | -13 |
| 102 | 14,15-LTE4 | 2.59 | 438/333 | -40 | -22 | -13 |
| 103 | 12-HETE | 11.49 | 319/135 | -50 | -19 | -13 |
| 104 | 12-HEPE | 9.16 | 317/179 | -30 | -19 | -13 |
| 105 | 14-HDoHE | 7.36 | 343/205 | -30 | -18 | -13 |
| 106 | 11-HDoHE | 12.38 | 343/149 | -20 | -19 | -13 |
| 107 | 9-HODE | 21.73 | 295.1/171 | -60 | -23 | -13 |
| 108 | HXA3 | 33.46 | 335/195 | -60 | -26 | -13 |
| 109 | HXB3 | 11 | 335/183 | -40 | -21 | -13 |
| 110 | 5-oxoETE | 11.21 | 317/203 | -60 | -22 | -13 |
| 111 | 12-oxoETE | 10.57 | 317/153 | -50 | -23 | -13 |
| 112 | 15-oxoETE | 33.48 | 317/113 | -20 | -25 | -13 |
| 113 | 9-oxoODE | 22.9 | 293/185 | -50 | -28 | -13 |
| 114 | 13-oxoODE | 11.58 | 293/167 | -50 | -29 | -13 |
| 115 | 15-oxoEDE | 1.9 | 321/223 | -80 | -32 | -13 |
| 116 | 20-HETE | 1.87 | 319/245 | -50 | -24 | -13 |
| 117 | 19-HETE | 10.57 | 319/231 | -40 | -23 | -13 |
| 118 | 18-HETE | 8.79 | 319/261 | -60 | -20 | -13 |
| 119 | 17-HETE | 2.12 | 319/247 | -50 | -20 | -13 |
| 120 | 16-HETE | 4.43 | 319/189 | -30 | -21 | -13 |
| 121 | 18-HEPE | 1.7 | 317/215 | -50 | -20 | -13 |
| 122 | 5,6-EET | 2.28 | 319/191 | -30 | -17 | -13 |
| 123 | 8,9-EET | 1.98 | 319/155 | -30 | -18 | -13 |
| 124 | 11,12-EET | 11.48 | 319/167 | -30 | -17 | -13 |
| 125 | 14,15-EET | 10.12 | 319/175 | -30 | -17 | -13 |
| 126 | 14(15)-EpETE | 6.96 | 317/207 | -30 | -19 | -13 |
| 127 | 17(18)-EpETE | 8.1 | 317/259 | -40 | -18 | -13 |
| 128 | 16(17)-EpDPE | 7.18 | 343/193 | -40 | -19 | -13 |
| 129 | 19(20)-EpDPE | 11.39 | 343/241 | -50 | -18 | -13 |
| 130 | 19,20-DiHDPA | 23.6 | 361/229 | -40 | -22 | -13 |
| 131 | 9,10-EpOME | 21.71 | 295/171 | -60 | -21 | -13 |
| 132 | 12,13-EpOME | 21.68 | 295/195 | -50 | -23 | -13 |
| 133 | 5,6-diHETrE | 2.08 | 337/145 | -40 | -22 | -13 |
| 134 | 8,9-diHETrE | 1.8 | 337/127 | -30 | -27 | -13 |
| 135 | 11,12-diHETre | 2.51 | 337/167 | -40 | -25 | -13 |
| 136 | 14,15-diHETrE | 23.84 | 337/207 | -30 | -24 | -13 |
| 137 | 9,10-diHOME | 12.23 | 313/201 | -50 | -29 | -13 |
| 138 | 12,13-diHOME | 18.86 | 313/183 | -50 | -29 | -13 |
| 139 | Arachidonic | 8.16 | 303/259 | -55 | -20 | -13 |
| 140 | Adrenic acid | 10.53 | 331/287 | -70 | -20 | -13 |
| 141 | EPA | 12.9 | 301/275 | -40 | -16 | -13 |
| 142 | DHA | 7.12 | 327/283 | -40 | -19 | -13 |
| 143 | 20cooh AA | 8.75 | 333/271 | -60 | -23 | -13 |
| 144 | 17k DPA | 23.62 | 343/247 | -40 | -23 | -13 |
| 145 | 2,3-dinor TXB2 | 8.94 | 341/137 | -20 | -31 | -13 |
| 146 | 11d-TXB2 | 1.91 | 367/305 | -20 | -26 | -13 |
| 147 | 2,3-dinor 8-iso PGF2a | 11.48 | 325/237 | -30 | -19 | -13 |
| 148 | 2,3-dinor-6k PGF1a | 10.21 | 363/281 | -30 | -23 | -13 |
| 149 | PGK1 | 12.82 | 351/251 | -40 | -26 | -13 |
| 150 | 8-iso PGF3a | 17.31 | 351/307 | -30 | -28 | -13 |
| 151 | 8-iso-15k PGF2b | 6.26 | 351/219 | -50 | -22 | -13 |
| 152 | 9-Nitrooleate | 1.9 | 326/168 | -40 | -20 | -13 |
| 153 | 10-Nitrooleate | 33.73 | 326/169 | -40 | -19 | -13 |
| 154 | tetranor-PGDM | 12.2 | 327/247 | -20 | -20 | -13 |
| 155 | 7(R)maresin-1 | 6.99 | 359/177 | -30 | -22 | -13 |
