# Supplementary material for: ER-depletion lowering the 'hypothalamus-uterus-kidney' axis functions by perturbing the renal ERβ/Ptgds signalling pathway
Source: Aging (Albany NY). 2019 Nov 10;11(21):9500–29. doi: 10.18632/aging.102401 (PMC6874469; doi:10.18632/aging.102401)
Supplement: Supplementary Table 3 [file aging-11-102401-s003.docx]

**Supplemental Table 3. Enrichment list of differentially expressed proteins on cellular component, cellular function, and biochemical process.** (cut-off valure 1.5-fold change, p value ˂ 0.05)

| **No.** | **Gene name** | **Abbreviation** | **Cellular Component** | **Cellular Function** | **Biochemical Process (GOID)** |
| --- | --- | --- | --- | --- | --- |
| 1 | Actin, cytoplasmic 1 | Actb | extracellular exosome(GO:0070062),  blood microparticle(GO:0072562),  membrane(GO:0016020),  focal adhesion(GO:0005925),  cytosol(GO:0005829),  MLL5-L complex(GO:0070688),  NuA4 histone acetyltransferase complex(GO:0035267),  cytoplasmic ribonucleoprotein granule(GO:0036464),  myelin sheath(GO:0043209),  cortical cytoskeleton(GO:0030863),  nuclear chromatin(GO:0000790) | ATP binding(GO:0005524),  RNA polymerase II core promoter proximal region sequence-specific DNA binding(GO:0000978),  RNA polymerase II distal enhancer sequence-specific DNA binding (GO:0000980) | substantia nigra development(GO:0021762),  platelet aggregation(GO:0070527),  ATP-dependent chromatin remodeling(GO:0043044) |
| 2 | Adenosine deaminase | Ada | Extracellular space(GO:0005615),  lysosome(GO:0005764),  cytoplasm(GO:0005737),  external side of plasma membrane(GO:0009897),  cell junction(GO:0030054),  cytoplasmic membrane-bounded vesicle lumen(GO:0060205),  neuronal cell body (GO:0043025),  dendrite cytoplasm (GO:0032839), | zinc ion binding (GO:0008270),  purine nucleoside binding (GO:0001883),  adenosine deaminase activity (GO:0004000) | Aging(GO:0007568),  positive regulation of T cell differentiation in thymus(GO:0033089),  liver development(GO:0001889),  adenosine catabolic process(GO:0006154),  negative regulation of mucus secretion (GO:0070256)，  negative regulation of circadian sleep/wake cycle, non-REM sleep(GO:0042323),  germinal center B cell differentiation(GO:0002314),  response to hypoxia(GO:0001666),  negative regulation of mature B cell apoptotic process(GO:0002906),  inosine biosynthetic process(GO:0046103),  dATP catabolic process(GO:0046061),  positive regulation of T cell receptor signaling pathway(GO:0050862),  positive regulation of germinal center formation(GO:0002636),  negative regulation of leukocyte migration(GO:0002686),  histamine secretion(GO:0001821),  T cell activation(GO:0042110),  negative regulation of thymocyte apoptotic process(GO:0070244),  adenosine metabolic process(GO:0046085),  negative regulation of inflammatory response(GO:0050728),  positive regulation of alpha-beta T cell differentiation(GO:0046638),  response to hydrogen peroxide(GO:0042542),  trophectodermal cell differentiation(GO:0001829),  lung alveolus development(GO:0048286),  positive regulation of calcium-mediated signaling(GO:0050850),  positive regulation of heart rate(GO:0010460),  positive regulation of smooth muscle contraction(GO:0045987),  positive regulation of B cell proliferation(GO:0030890),  placenta development(GO:0001890),  response to vitamin E(GO:0033197) |
| 3 | Alpha-2-HS-glycoprotein | Ahsg | extracellular space(GO:0005615),  blood microparticle(GO:0072562),  extracellular matrix(GO:0031012),  protein complex(GO:0043234), | cysteine-type endopeptidase inhibitor activity (GO:0004869),  endopeptidase inhibitor activity (GO:0004866),  receptor signaling protein tyrosine kinase inhibitor activity (GO:0030294),  kinase inhibitor activity (GO:0019210) | negative regulation of endopeptidase activity(GO:0010951),  acute-phase response(GO:0006953),  cellular response to insulin stimulus(GO:0032869),  negative regulation of cell growth(GO:0030308),  negative regulation of protein tyrosine kinase activity(GO:0061099),  cerebral cortex development(GO:0021987),  negative regulation of phosphorylation(GO:0042326),  regulation of inflammatory response(GO:0050727),  protein complex assembly(GO:0006461),  negative regulation of insulin receptor signaling pathway(GO:0046627) |
| 4 | Fructose-bisphosphate aldolase A | Aldoa | cytoplasm(GO:0005737),  mitochondrion(GO:0005739),  I band(GO:0031674),  M band(GO:0031430) | fructose-bisphosphate aldolase activity (GO:0004332) | response to hypoxia(GO:0001666),  glycolytic process(GO:0006096),  response to estrogen(GO:0043627),  protein homotetramerization(GO:0051289),  response to heat(GO:0009408), |
| 5 | Protein AMBP | Ambp | extracellular space(GO:0005615)  extracellular exosome(GO:0070062)  blood microparticle(GO:0072562)  plasma membrane(GO:0005886)  cell surface (GO:0009986)  intracellular membrane-bounded organelle(GO:0043231) | small molecule binding (GO:0036094), serine-type endopeptidase inhibitor activity (GO:0004867),  IgA binding (GO:0019862),  heme binding (GO:0020037),  protein homodimerization activity (GO:0042803) | negative regulation of endopeptidase activity(GO:0010951),  protein catabolic process(GO:0030163),  protein-chromophore linkage(GO:0018298) |
| 6 | Annexin A1 | Anxa1 | extracellular space(GO:0005615),  extracellular exosome(GO:0070062),  nucleus(GO:0005634),  plasma membrane(GO:0005886),  cytoplasm(GO:0005737),  focal adhesion(GO:0005925),  apical plasma membrane(GO:0016324),  lateral plasma membrane(GO:0016328),  extrinsic component of external side of plasma membrane(GO:0031232),  extrinsic component of endosome membrane(GO:0031313),  sarcolemma(GO:0042383),  protein complex(GO:0043234),  basolateral plasma membrane (GO:0016323),  mitochondrial membrane(GO:0031966),  phagocytic cup(GO:0001891),  mast cell granule(GO:0042629),  cornified envelope(GO:0001533),  motile cilium(GO:0031514),  early endosome membrane (GO:0031901),  cytoplasmic vesicle membrane (GO:0030659), | double-stranded DNA-dependent ATPase activity (GO:0033676),  phospholipase A2 inhibitor activity (GO:0019834),  calcium ion binding (GO:0005509), protein homodimerization activity (GO:0042803),  annealing helicase activity (GO:0036310),  structural molecule activity (GO:0005198),  single-stranded DNA binding (GO:0003697),  helicase activity (GO:0004386),  phospholipid binding (GO:0005543),  calcium-dependent phospholipid binding (GO:0005544) | lipase inhibitor activity (GO:0031960), regulation of lipase activity (GO:0060191), lipid transport (GO:0006869), fatty acid biosynthetic process (GO:0006633), response to glucocorticoid (GO:0051384), lipid localization (GO:0010876),  Regulation of interleukin-1 production(GO:0032652), response to organic cyclic compound (GO:0014070), inflammatory response (GO:0006954), DNA rewinding(GO:0036292), cell surface receptor signaling pathway (GO:0007166), estrous cycle (GO:0044849), positive regulation of neutrophil apoptotic process (GO:0033031), negative regulation of interleukin-8 secretion (GO:2000483), response to corticosteroid (GO:0031960), positive regulation of T-helper 1 cell differentiation (GO:0045627), granulocyte chemotaxis (GO:0071621), DNA strand renaturation (GO:0000733), regulation of leukocyte migration (GO:0002685), innate immune response (GO:0045087), positive regulation of prostaglandin biosynthetic process (GO:0031394), gliogenesis (GO:0042063), negative regulation of T-helper 2 cell differentiation(GO:0045629), response to hormone (GO:0009725), response to estradiol(GO:0032355), regulation of cell shape(GO:0008360), alpha-beta T cell differentiation(GO:0046632), prolactin secretion (GO:0070459), response to glucocorticoid (GO:0051384), response to peptide hormone (GO:0043434), regulation of hormone secretion(GO:0046883),cellular response to glucocorticoid stimulus(GO:0071385), positive regulation of vesicle fusion (GO:0031340), peptide cross-linking (GO:0018149), negative regulation of protein secretion (GO:0050709), regulation of inflammatory response (GO:0050727), adaptive immune response (GO:0002250), actin cytoskeleton reorganization (GO:0031532) positive regulation of T cell proliferation (GO:0042102), arachidonic acid secretion (GO:0050482), positive regulation of interleukin-2 production (GO:0032743), keratinocyte differentiation (GO:0030216), G-protein coupled receptor signaling pathway, coupled to cyclic nucleotide second messenger (GO:0007187), DNA duplex unwinding (GO:0032508), positive regulation of G1/S transition of mitotic cell cycle (GO:1900087), response to interleukin-1 (GO:0070555), insulin secretion (GO:0030073), monocyte chemotaxis (GO:0002548) |
| 7 | ATP synthase subunit beta, mitochondrial | Atp5b | mitochondrion(GO:0005739)  mitochondrial inner membrane (GO:0005743)  mitochondrial proton-transporting ATP synthase complex, catalytic core F(1)  (GO:0000275)  proton-transporting ATP synthase complex, catalytic core F(1) (GO:0045261)  mitochondrial proton-transporting ATP synthase complex(GO:0005753) | ATP binding (GO:0005524),  lipoprotein particle receptor activity (GO:0030228),  calcium ion binding (GO:0005509),  ATPase activity (GO:0016887),  proton-transporting ATP synthase activity, rotational mechanism (GO:0046933) | ATP metabolic process(GO:0046034),  receptor-mediated endocytosis(GO:0006898),  ATP hydrolysis coupled proton transport(GO:0015991),  ATP synthesis coupled proton transport(GO:0015986) |
| 8 | Beta-2-microglobulin | B2m | extracellular space(GO:0005615)  extracellular exosome(GO:0070062)  external side of plasma membrane  (GO:0009897)  focal adhesion(GO:0005925)  Golgi apparatus(GO:0005794)  MHC class I protein complex  (GO:0042612) |  | protein refolding (GO:0042026),  cellular response to lipopolysaccharide(GO:0071222),  positive regulation of T cell cytokine production (GO:0002726),  negative regulation of receptor binding(GO:1900121),  regulation of membrane depolarization(GO:0003254),  antigen processing and presentation of exogenous protein antigen via MHC class Ib, TAP-dependent(GO:0002481),  positive regulation of T cell mediated cytotoxicity(GO:0001916),  positive regulation of receptor-mediated endocytosis(GO:0048260),  T cell differentiation in thymus(GO:0033077) |
| 9 | Complement C3 | C3 | extracellular space(GO:0005615) | endopeptidase inhibitor activity (GO:0004866),  C5L2 anaphylatoxin chemotactic receptor binding (GO:0031715),  lipid binding (GO:0008289),  cofactor binding (GO:0048037) | complement activation, alternative pathway(GO:0006957),  blood coagulation(GO:0007596),  complement activation, classical pathway(GO:0006958),  inflammatory response(GO:0006954),  complement activation(GO:0006956),  tolerance induction(GO:0002507),  positive regulation of ERK1 and ERK2 cascade(GO:0070374),  regulation of triglyceride biosynthetic process(GO:0010866),  response to estradiol(GO:0032355),  positive regulation of developmental growth(GO:0048639),  response to glucocorticoid(GO:0051384),  positive regulation of protein phosphorylation(GO:0001934),  response to estrogen(GO:0043627),  positive regulation of glucose transport(GO:0010828),  positive regulation of lipid storage(GO:0010884),  chemotaxis(GO:0006935),  response to progesterone(GO:0032570),  positive regulation of G-protein coupled receptor protein signaling pathway(GO:0045745),  fatty acid metabolic process(GO:0006631),  response to magnesium ion(GO:0032026), |
| 10 | Cathelicidin antimicrobial peptide | Camp | extracellular space(GO:0005615)  cytoplasm(GO:0005737)  cell projection(GO:0042995) | cysteine-type endopeptidase inhibitor activity (GO:0004869) | positive regulation of cell proliferation(GO:0008284),  cellular response to lipopolysaccharide(GO:0071222),  cellular response to peptidoglycan(GO:0071224),  positive regulation of angiogenesis(GO:0045766),  positive regulation of protein phosphorylation(GO:0001934),  cellular response to tumor necrosis factor(GO:0071356),  cellular response to interleukin-1(GO:0071347)  cellular response to interleukin-6(GO:0071354) |
| 11 | CD44 protein | Cd44 | integral component of membrane  (GO:0016021) | hyaluronic acid binding (GO:0005540) | cell adhesion(GO:0007155) |
| 12 | Cd99 protein | Cd99 | integral component of membrane  (GO:0016021),  focal adhesion(GO:0005925) |  |  |
| 13 | Cadherin-1 | Cdh1 | integral component of membrane  (GO:0016021),  extracellular exosome(GO:0070062),  plasma membrane(GO:0005886),  cytoplasm(GO:0005737),  focal adhesion(GO:0005925),  lateral plasma membrane(GO:0016328),  aggresome(GO:0016235),  perinuclear region of cytoplasm (GO:0048471),  flotillin complex(GO:0016600),  cytoplasmic side of plasma membrane  (GO:0009898),  catenin complex(GO:0016342),  apical junction complex(GO:0043296),  endosome(GO:0005768),  cell-cell adherens junction(GO:0005913),  trans-Golgi network(GO:0005802),  lamellipodium(GO:0030027),  cortical actin cytoskeleton(GO:0030864) | calcium ion binding (GO:0005509) | positive regulation of transcription, DNA-templated(GO:0045893),  neuron projection development(GO:0031175),  homophilic cell adhesion via plasma membrane adhesion molecules(GO:0007156),  cellular response to indole-3-methanol(GO:0071681),  response to organic substance(GO:0010033),  single organismal cell-cell adhesion(GO:0016337),  positive regulation of transcription factor import into nucleus(GO:0042993),  negative regulation of cell-cell adhesion(GO:0022408),  synapse assembly(GO:0007416),  establishment of protein localization to plasma membrane(GO:0090002),  pituitary gland development(GO:0021983) |
| 14 | Collagen alpha-1(I) chain | Col1a1 | extracellular space(GO:0005615),  extracellular region(GO:0005576),  collagen type I trimer(GO:0005584),  Golgi apparatus(GO:0005794),  endoplasmic reticulum(GO:0005783),  secretory granule(GO:0030141) | metal ion binding (GO:0046872),  extracellular matrix structural constituent (GO:0005201) | protein transport(GO:0015031),  positive regulation of transcription, DNA-templated(GO:0045893),  positive regulation of cell migration(GO:0030355),  response to corticosteroid(GO:0031960),  response to estradiol(GO:0032355),  collagen biosynthetic process(GO:0032964),  response to cAMP(GO:0051591),  response to mechanical stimulus(GO:0009612),  response to peptide hormone (GO:0043434),  cellular response to tumor necrosis factor(GO:0071356),  osteoblast differentiation(GO:0001649),  bone trabecula formation(GO:0060346),  cellular response to mechanical stimulus(GO:00712600),  skin morphogenesis(GO:0043589),  negative regulation of cell-substrate adhesion(GO:0010812),  cartilage development involved in endochondral bone morphogenesis(GO:0060351),  cellular response to transforming growth factor beta stimulus(GO:0071560),  Ossification(GO:0001503),  response to hydrogen peroxide(GO:0042542),  response to steroid hormone(GO:0048545),  positive regulation of canonical Wnt signaling pathway(GO:0090263),  cellular response to amino acid stimulus(GO:0071230),  protein heterotrimerization(GO:0070208),  response to hyperoxia(GO:0055093),  cellular response to fibroblast growth factor stimulus(GO:0044344),  collagen fibril organization(GO:0030199),  blood vessel development(GO:0001568),  protein localization to nucleus(GO:0034504),  cellular response to epidermal growth factor stimulus(GO:0071364),  endochondral ossificationGO:0001958  positive regulation of epithelial to mesenchymal transition(GO:0010718),  embryonic skeletal system development(GO:0048706),  face morphogenesis(GO:0060325),  Response to nutrient (G0:0007584) |
| 15 | Ceruloplasmin | Cp | extracellular exosome(GO:0070062),  blood microparticle(GO:0072562),  lysosomal membrane(GO:0005765) | copper ion binding (GO:0005507),  ferroxidase activity (GO:0004322) | copper ion transport(GO:0006825),  cellular iron ion homeostasis(GO:0006879) |
| 16 | Protective protein for beta-galactosidase | Ctsa | lysosomal membrane(GO:0005765),  mitochondrion(GO:0005739) | serine-type carboxypeptidase activity (GO:0004185),  glycoprotein binding (GO:0001948) | Proteolysis(GO:0006508),  proteolysis involved in cellular protein catabolic process(GO:0051603),  regulation of protein stability(GO:0031647) |
| 17 | Dihydrolipoamide S-succinyltransferase (E2 component of 2-oxo-glutarate complex), isoform CRA_a | Dlst | extracellular exosome(GO:0070062),  nucleus(GO:0005634),  membrane(GO:0016020),  mitochondrion(GO:0005739),  oxoglutarate dehydrogenase complex  (GO:0045252),  myelin sheath(GO:0043209) | dihydrolipoyllysine-residue succinyltransferase activity (GO:0004149) | tricarboxylic acid cycle(GO:0006099) |
| 18 | Eukaryotic translation initiation factor 4A1 | Eif4a1 | extracellular exosome(GO:0070062),  cytoplasm(GO:0005737),  membrane(GO:0016020) | ATP binding (GO:0005524),  poly(A) RNA binding (GO:0044822),  ATP-dependent RNA helicase activity (GO:0004004),  double-stranded RNA binding (GO:0003725),  translation initiation factor activity (GO:0003743) | regulation of gene expression(GO:0010468),  RNA secondary structure unwinding(GO:0010501),  regulation of translational initiation(GO:0006446),  translational initiation(GO:0006413) |
| 19 | Ac2-120 | F5 | extracellular region(GO:0005576),  Golgi apparatus(GO:0005794),  endoplasmic reticulum(GO:0005783) | serine-type endopeptidase activity (GO:0004252),  copper ion binding (GO:0005507) | Proteolysis(GO:0006508),  blood coagulation(GO:0007596),  blood coagulation, extrinsic pathway(GO:0007598) |
| 20 | Lysosomal alpha-glucosidase | Gaa | Lysosome(GO:0005764),  lysosomal membrane(GO:0005765) | maltose alpha-glucosidase activity (GO:0032450),  alpha-1,4-glucosidase activity (GO:0004558),  carbohydrate binding (GO:0030246) | glycogen catabolic process(GO:0005980) |
| 21 | Gamma-glutamyl hydrolase | Ggh | extracellular space(GO:0005615),  extracellular exosome(GO:0070062),  nucleus(GO:0005634),  lysosome(GO:0005764),  cytosol(GO:0005829),  melanosome(GO:0042470) | gamma-glutamyl-peptidase activity (GO:0034722) | Proteolysis(GO:0006508),  response to ethanol(GO:0045471),  response to insulin(GO:0032868),  glutamine metabolic process(GO:0006541) |
| 22 | Gamma-glutamyltranspeptidase 1 | Ggt1 | extracellular space(GO:0005615),  plasma membrane(GO:0005886),  integral component of plasma membrane  (GO:0005887) | glutathione hydrolase activity (GO:0036374),  gamma-glutamyltransferase activity (GO:0003840) | Aging(GO:0007568),  peptide modification(GO:0031179),  glutathione catabolic process(GO:0006751),  response to lipopolysaccharide(GO:0032496),  response to estradiol(GO:0032355),  glutamate metabolic process(GO:0006536),  glutathione biosynthetic process(GO:0006750),  cellular response to oxidative stress(GO:0034599),  response to tumor necrosis factor(GO:0034612) |
| 23 | Beta-galactosidase | Glb1 | extracellular exosome(GO:0070062),  lysosome(GO:0005764),  Golgi apparatus(GO:0005794) | galactoside binding (GO:0016936),  beta-galactosidase activity (GO:0004565) | galactose catabolic process(GO:0019388),  cellular carbohydrate metabolic process(GO:0044262) |
| 24 | GM2 ganglioside activator | Gm2a | extracellular exosome(GO:0070062),  lysosome(GO:0005764),  cytoplasm(GO:0005737),  mitochondrion(GO:0005739),  cytoplasmic side of plasma membrane  (GO:0009898),  apical cortex(GO:0045179) | beta-N-acetylhexosaminidase activity  (GO:0004563),  phospholipase activator activity (GO:0016004),  lipid transporter activity (GO:0005319),  lipid binding (GO:0008289) | positive regulation of hydrolase activity(GO:0051345),  ganglioside metabolic process(GO:0001573),  ganglioside catabolic process(GO:0006689),  lipid transport(GO:0006869),  learning or memory(GO:0007611),  lipid storage(GO:0019915) |
| 25 | Heat shock 70 kDa protein 1-like | Hspa1l | blood microparticle(GO:0072562),  cytosol(GO:0005829),  mitochondrion(GO:0005739),  zona pellucida receptor complex  (GO:0002199),  mitochondrial matrix(GO:0005759),  COP9 signalosome(GO:0008180),  cell body(GO:0044297) | ATP binding (GO:0005524) | protein refolding(GO:0042026),  positive regulation of protein targeting to mitochondrion(GO:1903955) |
| 26 | Insulin-like growth factor binding protein 7, isoform CRA_b | Igfbp7 | extracellular space(GO:0005615),  extracellular exosome(GO:0070062),  extracellular matrix(GO:0031012) |  | response to organic cyclic compound(GO:0014070),  cell adhesion(GO:0007155),  response to cortisol(GO:0051414),  regulation of cell growth(GO:0001558),  response to heat(GO:0009408),  regulation of steroid biosynthetic process(GO:0050810),  cellular response to hormone  stimulus(GO:0032870), |
| 27 | Ig gamma-2B chain C region | Igh-1a | blood microparticle(GO:0072562),  external side of plasma membrane  (GO:0009897),  immunoglobulin complex, circulating  (GO:0042571) | antigen binding (GO:0003823),  immunoglobulin receptor binding (GO:0034987) | complement activation, classical pathway(GO:0006958),  positive regulation of B cell activation(GO:0050871),  B cell receptor signaling pathway(GO:0050853),  innate immune response(GO:0045087) |
| 28 | Immunoglobulin joining chain | Jchain | extracellular exosome(GO:0070062),  blood microparticle(GO:0072562),  secretory dimeric IgA immunoglobulin complex(GO:0071752),  pentameric IgM immunoglobulin complex(GO:0071756)  monomeric IgA immunoglobulin complex(GO:0071748),  dimeric IgA immunoglobulin complex  (GO:0071750) | immunoglobulin receptor binding (GO:0034987),  peptidoglycan binding (GO:0042834),  phosphatidylcholine binding (GO:0031210),  single-stranded DNA binding (GO:0003697) | innate immune response(GO:0045087),  positive regulation of protein oligomerization(GO:0032461),  adaptive immune response(GO:0002250),  glomerular filtration(GO:0003094),  humoral immune response(GO:0006959) |
| 29 | Leukemia inhibitory factor receptor | Lifr | integral component of membrane  (GO:0016021),  extracellular exosome(GO:0070062),  receptor complex(GO:0043235) | leukemia inhibitory factor receptor activity (GO:0004923),  oncostatin-M receptor activity (GO:0004924) | positive regulation of cell proliferation(GO:0008284) |
| 30 | Lipoprotein lipase | Lpl | extracellular exosome(GO:0070062),  plasma membrane(GO:0005886),  anchored component of membrane (GO:0031225),  extracellular matrix(GO:0031012),  cell surface(GO:0009986),  chylomicron(GO:0042627),  very-low-density lipoprotein particle  (GO:0034361) | triglyceride binding (GO:0017129),  lipoprotein lipase activity (GO:0004465), heparin binding (GO:0008201) | positive regulation of cholesterol storage(GO:0010886),  positive regulation of sequestering of triglyceride(GO:0010890),  lipid catabolic process(GO:0016042),  triglyceride biosynthetic process(GO:0019432)  triglyceride homeostasis(GO:0070328), acyl-glycerol metabolic process (GO:0046464), neutral lipid metabolic process (GO:0046461), triglyceride metabolic process (GO:0006641), fatty acid biosynthetic process (GO:0006633) |
| 31 | Lactoperoxidase (Predicted) | Lpo | extracellular space(GO:0005615),  extracellular exosome(GO:0070062),  cytoplasm(GO:0005737),  basolateral plasma membrane (GO:0016323) | thiocyanate peroxidase activity (GO:0036393),  heme binding (GO:0020037) | response to oxidative stress(GO:0006979) |
| 32 | Protein Lrp1 | Lrp1 | integral component of membrane (GO:0016021),  nucleolus(GO:0005730),  focal adhesion (GO:0005925),  dendrite(GO:0030425),  lysosomal membrane(GO:0005765),  receptor complex(GO:0043235),  neuronal cell body(GO:0043025),  endosome(GO:0005768),  clathrin-coated vesicle(GO:0030136) | poly(A) RNA binding (GO:0044822),  protease binding (GO:0002020),  calcium ion binding (GO:0005509) | Aging(GO:0007568),  cell proliferation(GO:0008283),  lipoprotein metabolic process(GO:0042157),  positive regulation of protein transport(GO:0051222),  cerebral cortex development(GO:0021987),  negative regulation of neuron projection development(GO:0010977),  protein kinase C-activating G-protein coupled receptor signaling pathway(GO:0007205) |
| 33 | Myosin-6 | Myh6 | Nucleoplasm(GO:0005654),  focal adhesion(GO:0005925),  myosin complex(GO:0016459),  stress fiber(GO:0001725),  Z disc(GO:0030018) | ATP binding (GO:0005524),  actin-dependent ATPase activity (GO:0030898),  motor activity (GO:0003774) | ATP metabolic process(GO:0046034),  regulation of heart rate(GO:0002027),  regulation of heart growth(GO:0060420),  adult heart development(GO:0007512),  ventricular cardiac muscle tissue morphogenesis(GO:0055010),  atrial cardiac muscle tissue morphogenesis(GO:0055009),  regulation of ATPase activity(GO:0043462),  cardiac muscle fiber development(GO:0048739),  BMP signaling pathway(GO:0030509),  canonical Wnt signaling pathway(GO:0060070),  cardiac muscle contraction(GO:0060048),  regulation of blood pressure(GO:0008217),  actin filament-based movement(GO:0030048) |
| 34 | Niemann Pick type C2 | Npc2 | extracellular exosome(GO:0070062),  lysosome(GO:0005764) | cholesterol binding (GO:0015485) | intracellular cholesterol transport(GO:0032367),  cholesterol homeostasis(GO:0042632),  cholesterol efflux(GO:0033344) |
| 35 | Plasminogen activator, urokinase | Plau | extracellular space(GO:0005615),  extracellular exosome(GO:0070062),  focal adhesion(GO:0005925),  cell surface(GO:0009986) | serine-type endopeptidase activity (GO:0004252),  kinase activity (GO:0016301) | response to lipopolysaccharide (GO:0032496),  Fibrinolysis (GO:0042730),  regulation of cell proliferation (GO:0042127),  response to hypoxia (GO:0001666),  regulation of receptor activity (GO:0010469),  regulation of cell adhesion mediated by integrin (GO:0033628) |
| 36 | Plasminogen | Plg | plasma membrane(GO:0005886),  extracellular region(GO:0005576),  intracellular membrane-bounded organelle(GO:0043231),  extrinsic component of plasma membrane (GO:0019897) | serine-type endopeptidase activity (GO:0004252),  endopeptidase activity (GO:0004175) | Fibrinolysis(GO:0042730),  tissue remodeling(GO:0048771),  blood coagulation(GO:0007596),  proteolysis involved in cellular protein catabolic process(GO:0051603),  labyrinthine layer blood vessel development(GO:0060716),  trophoblast giant cell differentiation(GO:0060707) |
| 37 | Anionic trypsin-2 | Prss2 | extracellular space(GO:0005615),  extracellular region(GO:0005576) | serine-type endopeptidase activity (GO:0004252),  calcium ion binding (GO:0005509) | Proteolysis(GO:0006508),  response to nutrient(GO:0007584),  collagen catabolic process(GO:0030574) |
| 38 | Prosaposin | Psap | extracellular exosome(GO:0070062),  lysosome(GO:0005764),  nucleoplasm(GO:0005654),  nucleolus(GO:0005730),  Golgi apparatus(GO:0005794),  mitochondrion(GO:0005739) |  | negative regulation of hydrogen peroxide-induced cell death(GO:1903206),  positive regulation of MAPK cascade(GO:0043410),  sphingolipid metabolic process(GO:0006665),  adenylate cyclase-inhibiting G-protein coupled receptor signaling pathway(GO:0007193),  cellular response to organic substance(GO:0071310),  regulation of lipid metabolic process(GO:0019216) |
| 39 | Prostaglandin-H2 D-isomerase | Ptgds | extracellular space(GO:0005615),  extracellular exosome(GO:0070062),  extracellular region(GO:0005576),  Golgi apparatus(GO:0005794),  perinuclear region of cytoplasm,  (GO:0048471),  rough endoplasmic reticulum,  (GO:0005791),  nuclear envelope(GO:0005635),  nuclear membrane(GO:0031965) | transporter activity (GO:0005215),  prostaglandin-D synthase activity (GO:0004667),  retinoid binding (GO:0005501),  fatty acid binding (GO:0005504) | response to glucocorticoid(GO:0051384), response to corticosteroid (GO:0031960), fatty acid biosynthetic process (GO:0006633),  prostaglandin biosynthetic process(GO:0001516) |
| 40 | Regenerating islet-derived protein 3-gamma | Reg3g | Cytoplasm(GO:0005737),  extracellular region(GO:0005576) | carbohydrate binding (GO:0030246) | acute-phase response(GO:0006953),  MyD88-dependent toll-like receptor signaling pathway(GO:0002755) |
| 41 | Lipase | RGD1565682 |  | hydrolase activity, acting on ester bonds (GO:0016788) | lipid catabolic process(GO:0016042) |
| 42 | Pre-eosinophil-associated ribonuclease-2 | Rnase2 |  | nucleic acid binding (GO:0003676),  ribonuclease activity (GO:0004540),  endonuclease activity (GO:0004519) | RNA phosphodiester bond hydrolysis(GO:0090501),  nucleic acid phosphodiester bond hydrolysis(GO:0090305) |
| 43 | RCG23287, isoform CRA_a | Rps27a | Ribosome(GO:0005840) | structural constituent of ribosome (GO:0003735) | Translation(GO:0006412) |
| 44 | Protein S100-A8 | S100a8 | extracellular space (GO:0005615),  extracellular exosome (GO:0070062),  nucleus (GO:0005634),  plasma membrane (GO:0005886),  cytoplasm (GO:0005737),  cytoskeleton(GO:0005856) | zinc ion binding (GO:0008270),  calcium ion binding (GO:0005509),  arachidonic acid binding (GO:0050544), antioxidant activity (GO:0016209) | response to lipopolysaccharide (GO:0032496), acute inflammatory response (GO:0002526),  chronic inflammatory response (GO:0002544),  inflammatory response (GO:0006954),  inflammatory response(GO:0006954),  innate immune response(GO:0045087),  positive regulation of peptide secretion(GO:0002793),  peptidyl-cysteine S-nitrosylation(GO:0018119),  positive regulation of inflammatory response(GO:0050729),  leukocyte migration involved in inflammatory response(GO:0002523),  activation of cysteine-type endopeptidase activity involved in apoptotic process(GO:0006919),  neutrophil chemotaxis(GO:0030593),  astrocyte development(GO:0014002) |
| 45 | Retinoid-inducible serine carboxypeptidase | Scpep1 | extracellular exosome(GO:0070062),  cytosol(GO:0005829) | serine-type carboxypeptidase activity (GO:0004185) | proteolysis involved in cellular protein catabolic process(GO:0051603),  positive regulation of vasodilation(GO:0045909),  negative regulation of blood pressure(GO:0045776) |
| 46 | Protein Sectm1b | Sectm1b | integral component of membrane  (GO:0016021) | signal transducer activity (GO:0004871) | signal transduction(GO:0007165),  immune response(GO:0006955) |
| 47 | Serine protease inhibitor A3K | Serpina3k | extracellular space(GO:0005615) | serine-type endopeptidase inhibitor activity (GO:0004867) | negative regulation of endopeptidase activity(GO:0010951) |
| 48 | Corticosteroid-binding globulin | Serpina6 | extracellular space(GO:0005615),  extracellular exosome(GO:0070062) | serine-type endopeptidase inhibitor activity (GO:0004867),  steroid binding (GO:0005496) | negative regulation of endopeptidase activity(GO:0010951),  glucocorticoid metabolic process(GO:0008211),  transport(GO:0006810) |
| 49 | Protein Serpinc1 | Serpinc1 | extracellular space(GO:0005615),  extracellular exosome(GO:0070062),  blood microparticle(GO:0072562) | serine-type endopeptidase inhibitor activity (GO:0004867),  heparin binding (GO:0008201) | response to lipopolysaccharide (GO:0032496),  response to estrogen (GO:0043627), negative regulation of endopeptidase activity(GO:0010951),  regulation of blood coagulation, intrinsic pathway(GO:2000266),  response to nutrient(GO:0007584),  negative regulation of inflammatory response(GO:0050728) |
| 50 | Protein Sh3bgrl3 | Sh3bgrl3 | extracellular exosome(GO:0070062),  lamellipodium(GO:0030027) | GTPase activator activity (GO:0005096), protein disulfide oxidoreductase activity (GO:0015035),  electron carrier activity (GO:0009055) | regulation of blood vessel endothelial cell migration(GO:0043535),  cell redox homeostasis(GO:0045454) |
| 51 | Neutral and basic amino acid transport protein rBAT | Slc3a1 | extracellular exosome(GO:0070062),  plasma membrane(GO:0005886),  integral component of plasma membrane  (GO:0005887),  mitochondrial inner membrane  (GO:0005743),  vacuolar membrane(GO:0005774)  brush border membrane(GO:0031526) | cation binding (GO:0043169),  protein heterodimerization activity (GO:0046982),  catalytic activity (GO:0003824) | carbohydrate metabolic process(GO:0005975),  amino acid transport(GO:0006865) |
| 52 | Extracellular superoxide dismutase [Cu-Zn] | Sod3 | extracellular space(GO:0005615),  extracellular exosome(GO:0070062),  nucleus(GO:0005634),  cytoplasm(GO:0005737),  extracellular matrix(GO:0031012),  trans-Golgi network(GO:0005802) | zinc ion binding (GO:0008270),  superoxide dismutase activity (GO:0004784),  copper ion binding (GO:0005507) | response to hypoxia(GO:0001666),  response to superoxide(GO:0000303),  response to oxidative stress(GO:0006979),  removal of superoxide radicals(GO:0019430),  response to copper ion(GO:0046688) |
| 53 | Trefoil factor 1 | Tff1 | extracellular space(GO:0005615),  cytoplasm(GO:0005737) |  | negative regulation of cell proliferation(GO:0008285),  cell differentiation(GO:0030154),  response to peptide hormone(GO:0043434),  response to immobilization stress(GO:0035902) |
| 54 | Protein Vnn1 | Vnn1 | extracellular exosome(GO:0070062) | pantetheine hydrolase activity (GO:0017159) | acute inflammatory response(GO:0002526),  positive regulation of T cell differentiation in thymus(GO:0033089),  chronic inflammatory response(GO:0002544),  innate immune response(GO:0045087),  single organismal cell-cell adhesion(GO:0016337),  negative regulation of oxidative stress-induced intrinsic apoptotic signaling pathway(GO:1902176),  central nervous system development(GO:0007417), |
| 55 | Protein Vtn | Vtn | extracellular space(GO:0005615),  cytoplasm(GO:0005737),  extracellular matrix(GO:0031012),  basement membrane(GO:0005604),  rough endoplasmic reticulum lumen  (GO:0048237),  Golgi lumen(GO:0005796) | polysaccharide binding (GO:0030247),  heparin binding (GO:0008201),  scavenger receptor activity(GO:0005044),  identical protein binding (GO:0042802),  collagen binding (GO:0005518) | immune responset(GO:GO:0006955),  protein polymerization(GO:0051258) |
